# Dissecting the Calcium-Induced Differentiation of Human Primary Keratinocytes Stem Cells by Integrative and Structural Network Analyses

**DOI:** 10.1371/journal.pcbi.1004256

**Published:** 2015-05-06

**Authors:** Kiana Toufighi, Jae-Seong Yang, Nuno Miguel Luis, Salvador Aznar Benitah, Ben Lehner, Luis Serrano, Christina Kiel

**Affiliations:** 1 EMBL/CRG Systems Biology Research Unit, Centre for Genomic Regulation (CRG), Barcelona, Spain; 2 Universitat Pompeu Fabra, Barcelona, Spain; 3 Institute for Research in Biomedicine, Parc Científic de Barcelona, Barcelona, Spain; 4 Institució Catalana de Recerca i Estudis Avançats, Barcelona, Spain; Brookhaven National Laboratory, UNITED STATES

## Abstract

The molecular details underlying the time-dependent assembly of protein complexes in cellular networks, such as those that occur during differentiation, are largely unexplored. Focusing on the calcium-induced differentiation of primary human keratinocytes as a model system for a major cellular reorganization process, we look at the expression of genes whose products are involved in manually-annotated protein complexes. Clustering analyses revealed only moderate co-expression of functionally related proteins during differentiation. However, when we looked at protein complexes, we found that the majority (55%) are composed of non-dynamic and dynamic gene products (‘di-chromatic’), 19% are non-dynamic, and 26% only dynamic. Considering three-dimensional protein structures to predict steric interactions, we found that proteins encoded by dynamic genes frequently interact with a common non-dynamic protein in a mutually exclusive fashion. This suggests that during differentiation, complex assemblies may also change through variation in the abundance of proteins that compete for binding to common proteins as found in some cases for paralogous proteins. Considering the example of the TNF-α/NFκB signaling complex, we suggest that the same core complex can guide signals into diverse context-specific outputs by addition of time specific expressed subunits, while keeping other cellular functions constant. Thus, our analysis provides evidence that complex assembly with stable core components and competition could contribute to cell differentiation.

## Author Summary

A key challenge in cellular network biology is to understand how protein complexes are cell-type or condition-specific assembled and disassembled. Cell differentiation is a major cellular reorganization bringing about fundamental changes in the new differentiated cell type. As many genes are expressed throughout all stages and only their expression levels differ, the question arises of how specific functions can be mediated. Here, focusing on the calcium-induced differentiation of primary human keratinocytes, we describe motifs of protein complex assemblies. We found that a large proportion of complexes contain both proteins expressed at similar levels in all stages of differentiation (non-dynamically expressed) and proteins with variable expression between (dynamically expressed). Using structural information we found that subunits tend to be replaced at structural overlapping surfaces of proteins. When applying our concepts to a manually annotated large TNF/NFkB signaling complex, we find a stable core associated with both a dynamically changing module and several stable modules. We propose this as a ‘constant signalosome ready to work,’ where a stable core is associated with a dynamic periphery. Altogether, our analysis highlights the importance of understanding the dynamic assembly and disassembly of complexes, taking 3D structural information into consideration, rather than only considering networks of individual proteins.

## Introduction

A key question in cellular network biology is how protein complexes assemble and disassemble in a time-dependent manner. Coordinated changes in the transcriptome and proteome occur during cellular differentiation [1–3], during cell reprogramming [4], or after growth factor stimulation [5] to name a few examples. Recent work in yeast has predicted that complexes change in composition during the cell cycle, and that complexes consist of both constitutive but non-dynamically expressed, and dynamically expressed subunits, leading to the proposal of ‘just-in-time assembly’ of complexes [6]. Consistent with this concept, relating expression data in different human cell types and tissues to protein complexes showed that non-dynamically expressed proteins extensively interact with tissue-specific expressed proteins, suggesting a tight interplay between core and tissue-specific proteins [7]. However, the molecular details underlying the assembly of complexes (‘complex assembly motifs’) are largely unexplored. This includes the definition of complexes themselves, e.g. as molecular machines (stably associated complexes) or pleiomorphic ensembles (complexes that assemble on demand) [8]. For example, what is the proportion of complexes that are permanently assembled, changed during different cellular conditions, or contain both non-dynamic and dynamic subunits (‘di-chromatic’ complexes)? Are subunits replaced at structurally overlapping or compatible surfaces of proteins? What is the role of evolutionarily-related paralogs?

Complementing protein interaction networks with three-dimensional structural information for binding interfaces has provided an improved functional understanding of cellular protein networks [9–15]. A recent study combining structural modeling with network analyses has revealed two types of interactions with a common hub protein: mutually exclusive interactions through a single interface (also called ‘XOR’) and compatible interactions through multiple interfaces (also called ‘AND’) [10]. This study has also shown that hub proteins characterized by single interfaces evolve faster and are enriched in paralogs [10]. In another study, modeling ErbB signaling through combining network and structural analyses, has suggested that competing protein interactions at single interface hubs produce variations in signaling responses [14].

Based on the above studies, we reasoned that the assembly of complexes where proteins compete for a common stable core could play a role in cell differentiation. To test this hypothesis and to define complex assembly motifs, we focused on the calcium-induced differentiation of primary human keratinocytes (PHK) as a model system for a large cellular reorganization process [16,17]. The epidermis of mammalian skin develops from a single layer of keratinocytes (interfollicular basal stem cells) into a multi-layered stratified epithelium. Keratinocyte differentiation is a well-suited model system, as differentiation of primary keratinocyte cells can be induced *in vitro* in cell culture by the addition of calcium [18]. In our previous work we quantified the transcriptome during differentiation, and we identified functionally important circadian oscillations [3]. Here, we performed a detailed analysis of all gene expression changes associated with keratinocyte differentiation followed during 45 hours and integrated this information with protein complexes to analyze their reorganization. We inferred that half of human protein complexes present during differentiation contain both non-dynamically and dynamically expressed subunits. Some di-chromatic complexes contain a stable core that associates with dynamic genes belonging both to similar clusters (concerted gene expression changes) and different clusters (opposing gene expression changes). In many cases, di-chromatic complexes with genes exhibiting opposing expression changes belong to complexes known to be involved in general or keratinocyte-specific differentiation processes and pathways, such as EGF/TGF-α signaling, TNF-α/NFκB signaling, Notch/γ-secretase, ubiquitination, cell cycle arrest, and chromatin remodeling complexes. Using three-dimensional structural modeling, we predicted physical interfaces and distinguished between mutually exclusive (XOR) and compatible (AND) interactions [13,19]. We found that dynamic proteins binding to a common non-dynamic protein are enriched for mutually exclusive interactions, suggesting that changes in complex assemblies can occur through variation in the abundance of proteins that compete for binding to XOR nodes. In addition, compensatory expression changes of paralogs suggest that these proteins—while keeping a constant essential function for cell viability—have differential functionalities, which serve a specific role for cell type-specific functions. Altogether our analysis highlights the importance of understanding the assembly of complexes and taking 3D structural information into consideration, rather than elucidating networks of individual proteins.

## Results

### Transcriptome profiling of the keratinocyte differentiation process identifies dynamic expression profiles

**Fig 1:**
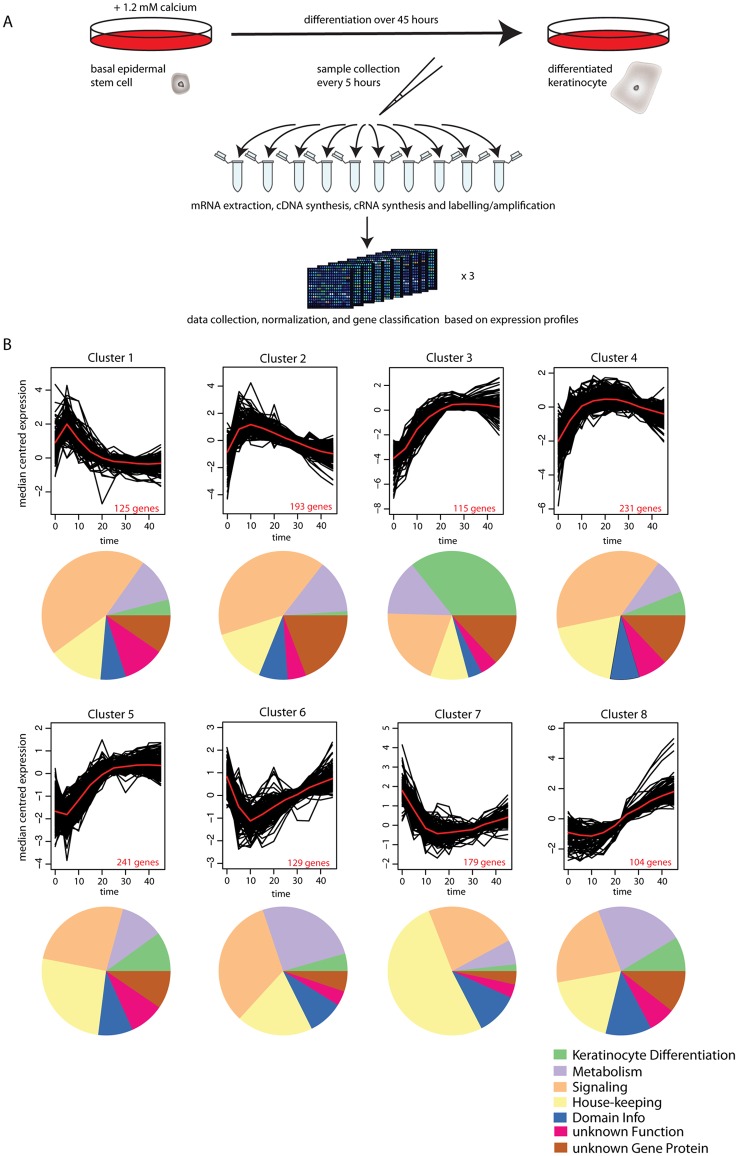
Transcriptome analysis of calcium-induced differentiation of human primary epidermal stem cells. (**A**) Experimental transcriptome analysis of the different process [3]. Differentiation was induced in human primary stem cells with 1.2mM CaCl2. Cells were collected every 5 hours in triplicates during 45 hours, total RNA was extracted from each sample, synthesized into cDNA, amplified in cRNA, Cy3 labeled, and hybridized to microarrays. Data were collected, normalized and gens were classified based on expression profiles. (**B**) Clusters of gene expression dynamics along differentiation. The 1317 expressed super-dynamic genes were clustered using K-means clustering and the proportion of the different functional categories is shown in the pie diagrams below. See also the legend.

Differentiation was initiated in human primary keratinocyte stem cells by the addition of CaCl_2_ [3, 18]. Cells were harvested over 45 hours at 5-hour intervals with three biological replicates. At each time point cells were lysed, mRNA was isolated, and expression profiles were measured using Agilent microarrays [3] (Fig 1A and S1 Table). 21,113 probes mapping to 16,720 genes were detected as expressed. We defined genes that are changing (‘dynamic’) and those that are constantly expressed (‘non-dynamic’) using a 2-fold expression change cut-off and a Chi-squared test on time points of biological replicates (see methods). As a more stringent criterion for classifying dynamic genes, we used a threshold of 4-fold, defining these genes as ‘super-dynamic’ (S1 Table). In summary, out of the 16,720 expressed genes, 6,137 are ‘non-dynamic’ (P> = 0.01, Chi-squared test, and fold change <2), 6,096 are ‘dynamic’ (P<0.01 and fold change > = 2), 1,317 of the dynamic genes are also ‘super-dynamic’, (P<0.01 and fold change > = 4), and 4,487 genes do not fall into one of the above categories (classified as ‘unresolved’). In general, we used the super-dynamic genes for our analyses, unless otherwise stated.

Classification of proteins encoded by the super-dynamic genes based on DAVID [20,21], UniProt [22], and manual literature searches (e.g. [23]) (S1 Fig) uncovered a large fraction of proteins involved in: i) signaling (transcription factors, and the adhesion, chemokine, calcium, immune/Toll, ubiquitin-like, apoptosis, Wnt, TGFβ, interferon, and Notch signaling pathways (S2 Fig) (32%); ii) housekeeping functions (cytoskeleton, cell cycle, solute transport carriers, histone, and chaperone proteins, or formation of tight junctions (S3 Fig) (22%); iii) metabolic enzymes for lipid, amino acid, steroid, purine, and flavine interconversions and lipid binding (S4 Fig) (13%), iv) proteins needed for the progressive steps towards the formation of the cornified envelope (metallo and serine proteases), crosslinking enzymes (e.g. transglutaminases) and substrates (e.g. loricrin, involucrin, and small proline rich proteins) that provide structural stability and elasticity, keratins (mechanical resistance), and lipid modifying enzymes (water repellence) (S5 Fig) (8%) [23]. For 8% of the super-dynamic genes only one Pfam domain prediction can be assigned, while 17% of them have no known function or Pfam domain annotations. Thus, the process of keratinocyte differentiation is accompanied by concerted changes in metabolic, signaling, and housekeeping pathways. We also confirmed the induction of known markers for keratinocyte differentiation (involucrin [IVL], filagrin [FLG], cystatin [CSTA, CSTB]) (S1 Table) [23]. Involucrin expression was additionally confirmed on the protein level by immunostaining (S6 Fig).

### Unsupervised clustering partitions the temporal profiles of super-dynamic genes into eight clusters

We used K-means clustering to classify the temporal profiles of the 1,317 super-dynamic genes and identified eight optimized clusters (Fig 1B). The clusters are arranged with opposing temporal profiles on the top and on the bottom: clusters 1 and 5 contain early highly transient expressed/repressed genes, clusters 2 and 6 contain early transient expressed/repressed genes, clusters 3 and 7 contain early highly sustained expressed/repressed genes, and clusters 4 and 8 contain early sustained expressed/repressed genes. Consistent with the function of the Ets transcription factor ELF3 in promoting differentiation of keratinocytes [24,25], we see strong immediate and sustained expression of ELF3 (cluster 3), followed by delayed expression of genes regulated by ELF3 like KRT4 and TGFβ (cluster 8), and the envelope protein SPRR2A (cluster 3) [26,27].

The clusters reveal a moderate stage-specific expression of functionally related protein classes and biological processes. For instance, proteins related to the formation of the cornified envelope are strongly induced at later time points (clusters 3, 4, 5, and 8). Cluster 7 (repressed genes) is enriched for cell cycle-related proteins in the housekeeping category, as expected during the onset of differentiation [28]. This cluster has an antagonistic behavior when compared to the cell-cycle enriched cluster published in a cellular reprogramming study [4]. Signaling-related pathways in clusters 1 and 2 involve transiently expressed transcription factors, cytokines, and proteins involved in Wnt, adhesion, TGFβ, and TNF signaling (S1 Table). Likewise, enzymes involved in lipid and amino acid metabolism are present in most clusters. The absence of a strong association between functional categories and clusters suggests a functional replacement within sub-categories, possibility brought about in some cases by dynamic rearrangements within protein complexes.

**Fig 2:**
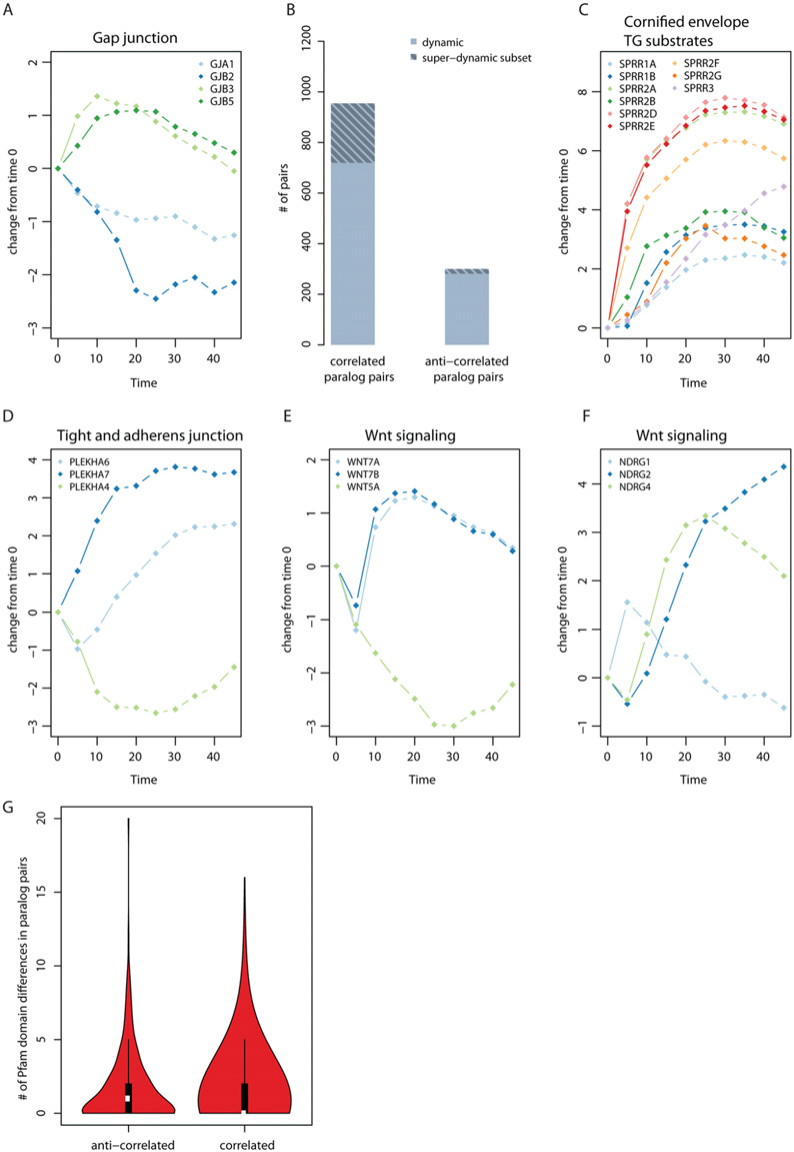
Paralog analysis of dynamic genes. (**A**) Known example from literature for opposite expression changes of paralogs involved in gap junction permeability [29]. (**B**) Statistics for correlated and anti-correlated dynamic and super-dynamic proteins. There are 950 highly co-expressed (r> = 0.6) dynamic paralogous pairs, 235 of which are super-dynamic and 281 highly anti-correlated (r< = -0.6) dynamic paralogous pairs, 19 of which are super-dynamic. (**C**) Example for correlated pairs (super dynamic), grouped by paralogous families. (**D**), (**E**), and (**F**): example for anti-correlated (super dynamic) grouped according to paralogous families. (**G**) Pfam domain differences between dynamic paralogous pairs. 281 anti-correlated paralogs (r< = -0.6) have a mean of 1.7 differences compared to 950 correlated paralogs (r> = 0.6) which have a mean difference of 1.28 in their domains (P = 0.009). The statistical significance was tested using Wilcoxon rank sum test.

During keratinocyte differentiation, a switch in the expression of paralogous gap junction subunits is needed for the changes in gap junction permeability required for epidermis formation [29], which we see in our study (Fig 2A). To see if this is a general case for all paralogous pairs, we annotated expressed genes in keratinocytes with homology information from EnsemblCompara [30] limiting our analysis to paralogous pairs in which both genes are dynamically expressed, thereby reducing our set from 11,582 to 2,260 paralogous pairs. Computing Pearson correlation metric for all pairs, we found 950 to have highly correlated (r> = 0.6, P<0.07) dynamic expression profiles during skin differentiation with a subset of 235 qualifying as super-dynamic, and 281 pairs to have highly anti-correlated dynamic expression (r< = -0.6, P<0.07) with 19 of these pairs being super-dynamic (Fig 2B and S2 Table). When compared to super-dynamic and dynamic random pairs, we found that paralagous pairs are more likely to have correlated expression (Wilcoxon rank sum test; P = 4.5e-37, for super-dynamic S7A Fig; and P = 5.4e-61 for dynamic, S7B Fig). An example where gene duplicates can act together to bring about a functional change is the formation of cornified envelope (e.g. the transglutaminase substrates, small proline-rich proteins) (Fig 2C and S8 Fig). This could suggest a requirement for increased gene dosage in particular specific stages of cell differentiation as the likely reason for the duplication of these genes [31–33].

We also found nineteen anti-correlated pairs where both proteins are super-dynamic (Figs 2D–2F and S9 Fig). Examples include PLEKHA 6/7/4 (Fig 2D; for PLEKHA-7 a functional role has been demonstrated in recruiting paracingulin to tight junctions [34]), WNT7A/7B/5A (Fig 2E), NDRG1/2/4 (Fig 2F; NDRG2 is expressed in response to TGFβ and inhibits proliferation [35]) and CCNA/B proteins (S9 Fig). One explanation for this observation might be that there is temporal sub-functionalization, with one paralog being expressed early, the other late during the course of differentiation as the paralogs have taken on different functions. Therefore, the expectation would be that anti-correlated paralogous pairs are more divergent in sequence level than correlated ones. Using Pfam domain annotations we compared correlated to anti-correlated dynamic paralogous pairs (here we used dynamic pairs to increase the numbers for statistical analysis since not every protein has Pfam annotations) and found that correlated paralogous pairs are more similar in their Pfam domain composition (different domain, or domain missing) than anti-correlated pairs (1.3 versus 1.7; Wilcoxon rank sum test; P = 0.009) (Fig 2G and S10 Fig). Likewise, anti-correlated dynamic paralogous pairs have greater sequence divergence (S11A and S11B Fig) and a greater number of differences in amino acid sequence length (S11C and S11D Fig). Furthermore, anti-correlated genes have a higher fraction of evolutionary old duplicated genes compared to correlated genes (S12 Fig).

In summary, dynamic paralogs are enriched in gene pairs that are correlated in expression. This suggests that a requirement for increased gene dosage is the likely reason for the duplication of these genes. Paralogous genes with anti-correlated expression changes have greater amino acid sequence and Pfam domain divergence. This implies that anti-correlated paralogs have taken on specialized roles during differentiation.

### Expression changes reveal dynamically expressed genes encoding proteins in complex with proteins encoded by non-dynamically expressed genes

**Fig 3:**
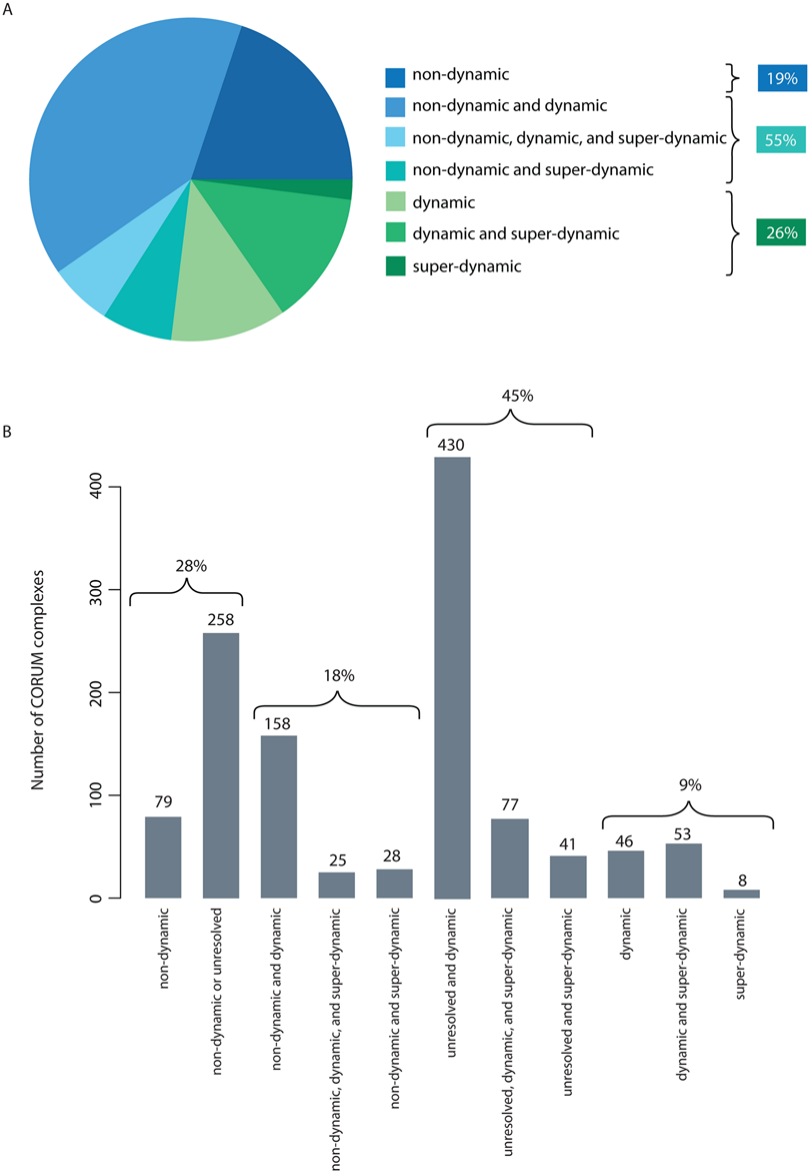
Statistics of expression profiles mapped on human protein complexes. (**A**) Statistic of protein complexes with subunits classified as non-dynamic, unresolved, dynamic, super-dynamic, or a combination of classes. When including unresolved genes in the non-dynamic class, 28% of the complexes are non-dynamic/unresolved, 18% are di-chromatic (63% if we include unresolved genes), and 9% are only dynamic/super-dynamic. (**B**) Excluding all complexes with any unresolved subunits results in 55% di-chromatic complexes, 19% completely non-dynamic, and 26% completely dynamic/super-dynamic.

**Fig 4:**
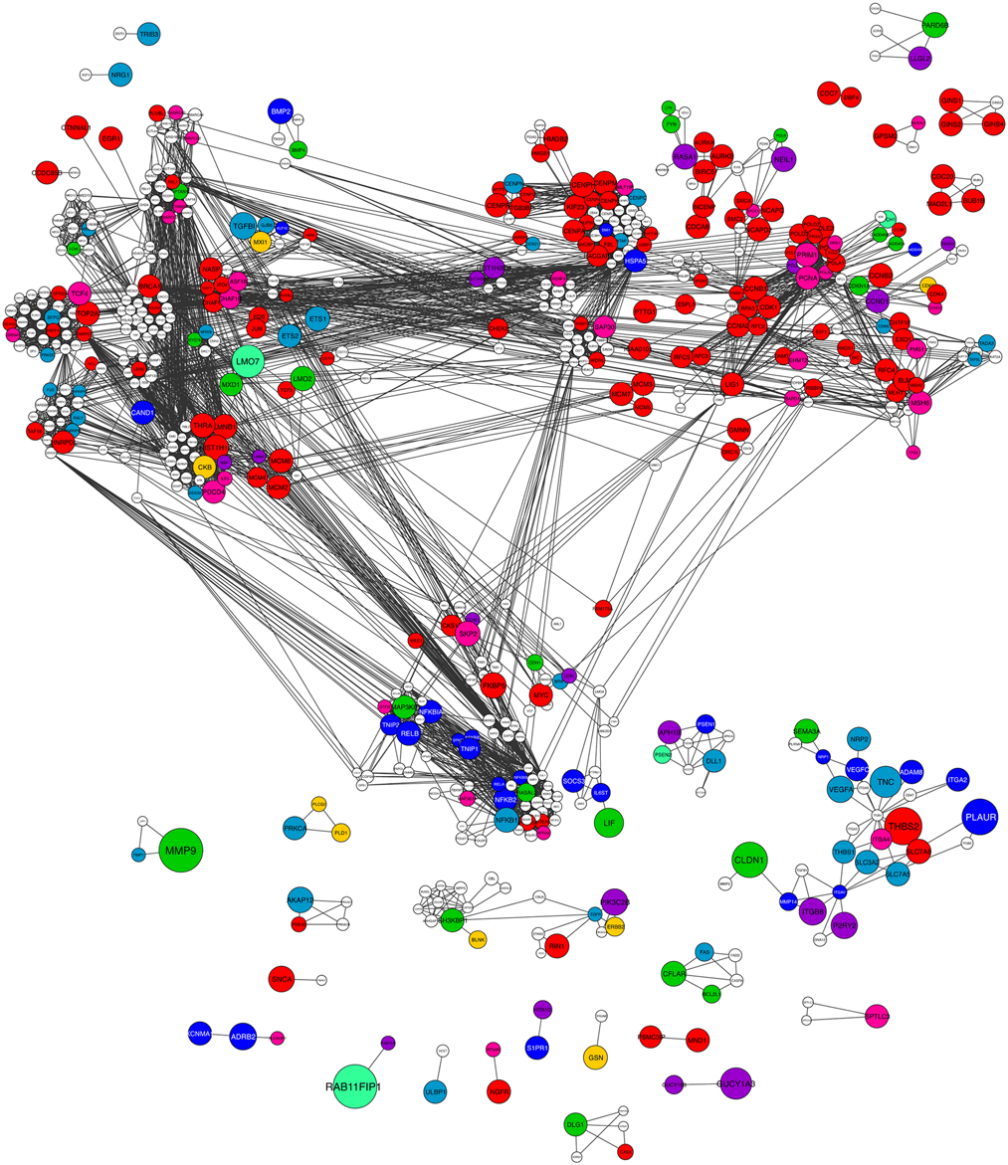
Network representation of human protein complexes with di-chromatic expression profiles. Protein interaction network representation of human protein complexes containing at least one super-dynamic subunit. Dynamic genes were re-clustered based on best agreement with one of the eight super-dynamic clusters. The nodes are colored according to cluster membership (see Fig 5) and node sizes are indicative of fold absolute changes over time. The network is represented using Cytoscape.

The finding that divergent paralogous pairs tend to have anti-correlated expression patterns could suggest replacement of subunits in protein complexes to expand functional complexity. To see if this is a general feature of protein complexes during differentiation, we obtained a list of protein complexes from the CORUM database (a resource of manually annotated protein complexes from mammalian organisms [36]), and mapped it onto the non-dynamically expressed, unresolved, or dynamically changing proteins (Fig 3A and S13 Fig and S3 Table). After removing complexes containing unresolved genes, 19% of the complexes did not have significantly changing gene expression for any of their subunits during the keratinocyte differentiation process (all non-dynamic), 26% of the complexes had dynamically changing expression for all subunits, and for half of the complexes (55%) we find a mix of behaviors (here called ‘di-chromatic’) (Fig 3B). We represented the previously clustered 1,317 super-dynamic genes in the context of complexes to which they belong and also assigned dynamic genes to each one of the eight super-dynamic clusters through comparative correlation analysis with average expression profile of the aforesaid clusters (Fig 4; S1 Network). We found that super-dynamic genes in the same complex or closely connected in the network often belong to different clusters (72%). However, this ratio decreases to 43% if clusters with similar behaviors (1 and 2, 3 and 4, 5 and 8, 6 and 7) are combined.

**Fig 5:**
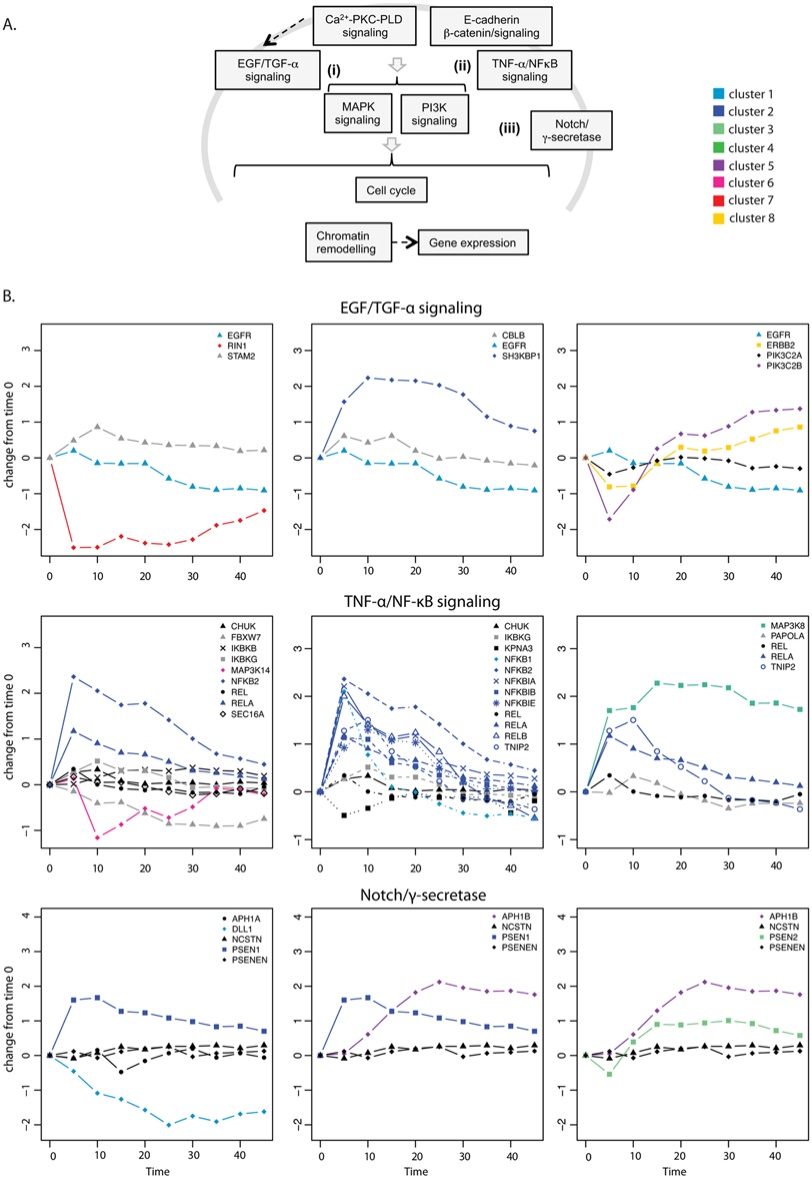
Selected di-chromatic complexes with cellular functions involved in keratinocyte differentiation. (**A**) Schematic overview of cellular processes involved in keratinocyte differentiation. (**B**) Selected CORUM complexes of similar core proteins were plotted. Super-dynamic and dynamic genes were colored according to clusters. Black indicates constitutive genes, grey indicated unresolved genes.

To further characterize the expression profiles of di-chromatic complexes, we classified the CORUM complexes into functional groups (S3 Table). Then we represented the expression changes for functionally related complexes or complexes sharing components (Fig 5 and S14–S16 Figs). Among many cellular processes known to be important for keratinocyte differentiation or general cell differentiation (Fig 5A), we find examples of complexes with a stable component or core and a dynamic periphery. Examples in cell signaling include EGFR and TNF-α/NFκB pathways. EGF signaling is important for keratinocyte differentiation [37] and we find two complexes involved in its recycling and degradation. In both complexes, one component (STAM2 and CBLB) is constant and the other is super-dynamic (RIN1 and SH3BKP1, respectively), changing in opposite directions in the two complexes. RIN1 regulates EGFR degradation in cooperation with STAM [38], and SH3BKP1 prevents epidermal growth factor receptor degradation by the interruption of c-Cbl-CIN85 complex [39]. Thus, the opposite behavior of these complexes will favor EGFR stabilization and consequently, EGF signaling and keratinocyte differentiation. In this respect we also find anti-correlated changes of the paralogous receptors EGFR and ErbB2, which slow down EGFR recycling through heterodimer formation [40].

The TNF-α/NF-κB signaling pathway is required for normal epidermal development and homeostasis [41–43]. As the CORUM database is not complete [44], we combined the CORUM complex information with a detailed structural and pathway analysis based on the literature [45, 46] (S15 Fig). We identified a core TNF receptors/scaffold complex (TNFRSF1, TRADD, RIPK1, TRAF2, IKBG1, CHUK, CDC37 and HSP90AA1) associated with a MAPK pathway (i.e. MAP2K7/ and MAPK8/9) which is composed of non-dynamic and unresolved genes, while the rest of the pathway in general is composed of dynamic components. This is an example of a stable core module associated with a dynamic peripheral module. Most of the super-dynamic genes are found mainly in the transiently expressed clusters 1 and 2, suggesting relevant concerted changes during keratinocyte differentiation (Fig 5B). MAP3K8 (Cot) is in cluster 4 (early activated and sustained expression) and can form larger complexes containing NFKB1, thereby promoting signaling. Likewise, CFLAR is in the same cluster 4 and is an inhibitor of FADD thereby preventing apoptosis. NF-κB/RelA has been shown to control cell-cycle exit in keratinocytes [47].

NF-κB-independent signaling commencing at the level of the non-dynamic core connects to MAPKs (such as NIK, NAK, TAK1, and Cot) and PKC isoforms [48]. Interestingly we see that TRAF1 expression is in the same cluster as the NF-κB components. It has been described that TRAF1 promoter has NF-κB binding sites and is strongly activated by TNF-α [49]. TRAF1 cannot bind to the TNF receptor directly, but is recruited though binding to TRAF2 [50] and overexpressing TRAF1 does not affect the interaction between TRAF2 and FADD [51], suggesting a compatible complex formation. TRAF1 binding to TRAF2 results in blocking apoptosis [52] and analysis of TRAF1-/- mice suggests that TRAF1 inhibits TNF-α signaling [49]. Thus, TNF-α/NF-κB induced dynamic expression of TRAF1 creates a negative feedback loop that could mediate anti-apoptotic functions [52] and decrease NF-κB activation without affecting other possible constitutive function of TNF receptor. We propose that the stable core module connects to both a dynamic peripheral module important for cell cycle arrest during differentiation (via NF-κB heterodimers and prevents apoptosis (via TRAF1), and several stable modules that function independent of NF-κB by binding to TRAF or the IKK complex and which should play a housekeeping function [48] (see S15 Fig for a summary of different signaling functions).

In the Notch/γ-secretase pathway [53], the γ-secretase (APH1B, promoting differentiation) is up-regulated and delta like 1 (DLL1, blocking differentiation) is down-regulated (Fig 5B). Both proteins associate to the same stable core (PSENEN and NCSTN), reinforcing a biological role for di-chromatic complexes. In the ubiquitination/degradation pathway, the CAND1 assembly factor of the E3 ubiquitin ligase complex [54] is up-regulated transiently and the S-phase kinase-associated protein 2 (SKP2, promoting cell cycle) is down-regulated as the target of the E3 ubiquitin ligase complex (S14A Fig). Skp2–Skp1 abrogates the inhibitory influence of CAND1 on the neddylation of Cul1 by promoting the dissociation of the cullin–CAND1 complex, whereas substrate, together with substrate-presenting components, prevents the action of CSN to deneddylate cullin [55]. It has been described that high levels of Skp2 are needed for proliferation in stratified epithelia [56]. In this respect it is noteworthy that CAND1 also causes elevation of p27, which has been demonstrated to be important during pre-adipocyte differentiation [57].

Finally, two complexes with interesting di-chromatic behavior are related to chromatin remodeling and known to be important for regulating gene expression changes during keratinocyte differentiation [58,59]. Emerin is a nuclear membrane protein, which is involved in tissue-specific gene regulation [60] and expressed constantly during the keratinocyte differentiation (S14C Fig). The emerin binding protein LMO7 is a cell type-specific transcription factor that is strongly up-regulated during differentiation. It acts by escaping actin (ACTB)-mediated inhibition [61,62] and therefore its levels need to increase strongly. At the same time, Laminin B1 (LMNB1; important for DNA replication) is transiently down-regulated. Finally, the BAF (SWI/SNF) complexes are known for their combinatorial assembly providing functional specificities [63,64] (S14D Fig). BRCA1 can directly interact with the BRG1 subunit of the SWI/SNF complex and is down-regulated during differentiation. This may liberate the SWI/SNF complexes to take part in chromatin remodeling, which are either constantly expressed or moderately down-regulated.

Generalizing, there are three different types of assembly motifs: In some complexes, dynamic genes are added or removed from the complexes and these are predicted to be compatible (AND; [14]) interactions (e.g. TNF-α/NFκB signaling and Notch complexes). For other complexes we observed opposing expression changes of subunits, which are potentially competing for the same binding interface (XOR interactions; i.e. CULIN SKP2 CAND1 [14]). Finally, there are large assemblies where we observed a mixed behavior (i.e. EGFR/TGF-β; S16 Fig). In summary, we find a di-chromatic behavior with a stable core for many complexes involved in cell differentiation. We suggest that the different assembly motifs with respect to compatible (AND) and mutually exclusive (XOR) surface interactions should be classified using 3D structural information to analyze which type of assembly motifs dominate during keratinocyte differentiation.

### Structural analyses identify mutually exclusive surface interactions as enriched in dynamic genes

**Fig 6:**
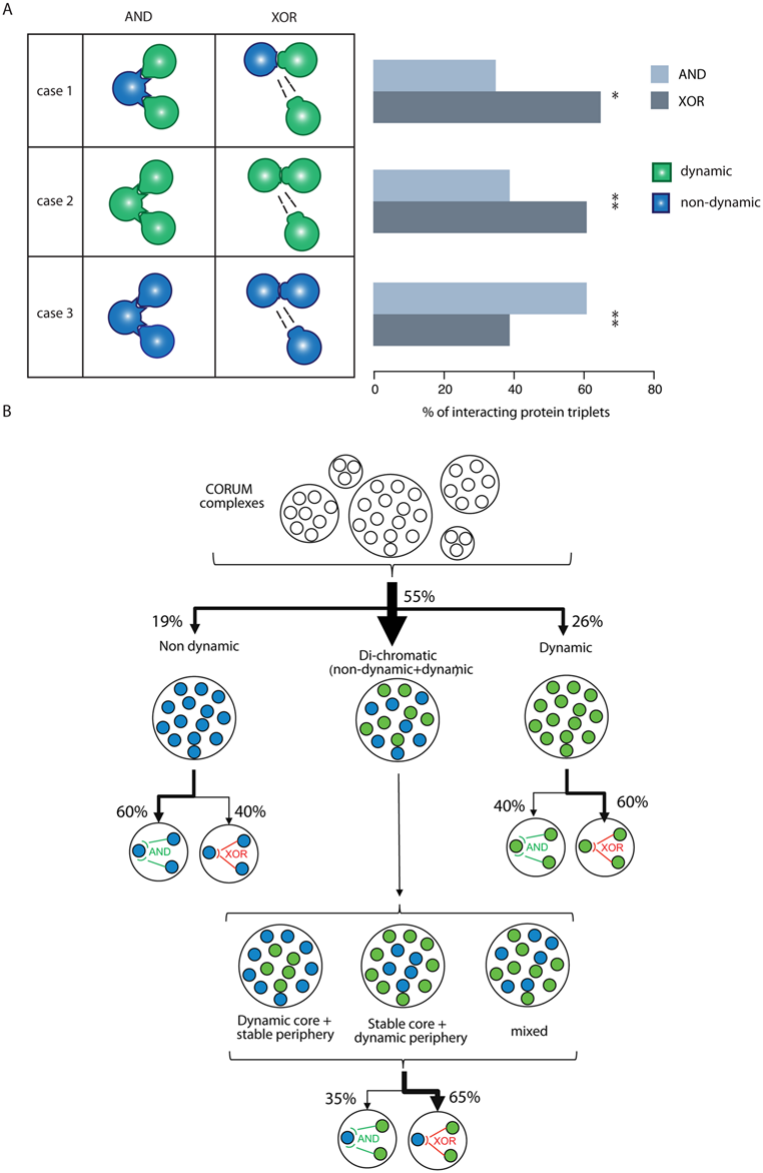
Combining expression classification with compatible (AND) and mutually exclusive (XOR) interaction types and summary of complex assembly motifs. (**A**) Left panel, schematic representation of possible cases combining protein expression classification with surface interaction types: compatible (AND) and mutually exclusive (XOR) interactions. Discounting unresolved class, there are six possible cases, out of which three biologically meaningful ones were selected. Case 1: the hub protein is non-dynamically expressed while the attachment proteins are dynamic; case 2: all three interacting proteins are dynamic; and case 3: all three interacting proteins are non-dynamic. For the remaining cases, including unresolved genes, see S16 Fig Right panel shows percentages of protein interaction triplets analysed structurally by SAPIN for cases 1 to 3. Of protein triplets of case 1, 65% (n = 49) have XOR interactions compared to 35% (n = 27) AND, of case 2 61% (n = 128) have XOR interactions compared to 39% (n = 81) AND interactions and finally of case 3, 61% show AND interactions (n = 628) compared to 39% (n = 397) XOR. Statistical significance tested using Fisher test where * indicates p < 0.0001 and ** indicates p < 0.00001. Multiple testing correction was conducted with Bonferroni method resulting in all q < 0.005. For the remaining cases, including unresolved genes, see S19–S21 Figs (**B**) Summary of the complex assembly motifs proposed for the process of keratinocyte differentiation.

Proteins that bind mutually exclusively to the same domain on a shared binding partner protein prevent each other’s binding through steric hindrance depending on concentration and localization [10, 13, 14]. Steric hindrance and competition could be a mechanism to achieve cell type-specific functions if a competing protein is expressed at a higher level in a specific cell type or tissue [14]. To determine if the replacement of subunits of complexes during the differentiation process happens at mutually exclusive surface interactions, we structurally analyzed all CORUM complexes with less than 20 members using the SAPIN software framework (S17 Fig) [19]. SAPIN identifies the protein regions that could be involved in an interaction, provides template structures, and then performs structural superimpositions to identify compatible and mutually exclusive interactions. If a protein has at least two interacting partners, the domains mediating the interaction are superimposed on the reference domain, and the interacting domains are analyzed for compatibility (AND) or mutual exclusiveness (XOR) (S4 Table). Next, we combined expression classification during keratinocyte differentiation (non-dynamic vs. dynamic) with compatible and mutually exclusive interaction types (S18 Fig). Out of six possible cases (obviating the unresolved group), three cases were selected that we could interpret in terms of competition (Fig 6A). In case 1, the hub protein (common protein with at least two different binding partners) is non-dynamically expressed while the attachment proteins are dynamic. In case 2, all three interacting proteins are dynamic. In case 3 all three interacting proteins are non-dynamic. Interestingly, case 1 is significantly enriched for gene products with mutually exclusive surface interactions (XOR) (61% compared to 39%, Fisher’s exact test; P = 1.9e-6), reinforcing our hypothesis that dynamic genes tend to be involved in competing interactions for a common (constitutively non-dynamically expressed) binding partner (Fig 6 and S19–S21 Figs). Case 3 is significantly enriched for AND (61% compared to 39%, Fisher’s exact test; P = 2.03e-12), representing stable complexes that do not change their composition during the differentiation process. Case 2 (all three proteins dynamically changing) is also enriched for XOR interactions compared to AND (65% compared to 35%, Fisher’s exact test; P = 8.66e-04). When focusing on the 32 XOR nodes with at least two super-dynamic genes, 26 XOR nodes contain genes from at least 2 different clusters suggesting that opposing or at least different expression profiles may impose different functional outputs possibly to compete at single interface hubs. Thus, variation in the concentration of proteins (belonging to different clusters) that bind to XOR nodes may cause complex re-assembly through competition and thus achieve a different functional output in the differentiated keratinocytes or during the process of differentiation.

## Discussion

Using calcium-induced differentiation of primary keratinocytes as a model system for a substantial cellular re-organization, we analyzed transcriptome changes during 45 hours. We discovered a large proportion of genes change their expression during the differentiation process (36% dynamic and 7.9% super-dynamic genes), which is comparable with other cell differentiation or reprogramming studies [2,4, 65]. Our set of dynamically up-regulated genes contains 39 out of 53 previously described keratinocyte differentiation markers, 25 of which are super-dynamic [66]. The super-dynamically changing genes, aside from those with known keratinocyte function, span metabolism [67], signaling, and housekeeping cellular functions. Interestingly a quarter of super-dynamic genes are of unknown functions based on UniProt annotations and manual literature searches. The super-dynamic genes were partitioned into eight clusters, with only those genes needed for establishing the final physiological functions of the cornified envelope (e.g. water repellence, structural stability, and mechanical resistance) exhibiting a clear GO-enrichment. Many metabolic, signaling and housekeeping genes were found in all clusters. Interestingly, the shapes of the clusters are similar to those observed in other cell differentiation or reprogramming studies [4,65]. Yet, due to an absence of strong enrichment in GO terms in this study and others, direct comparison between the functions of the clusters could not be conducted. Both correlated and anti-correlated paralogous pairs have been discovered before, during reprogramming of somatic cells to pluripotency [4]. Similarly, we found that dynamic paralogs are enriched in correlated expression changes, which may represent examples of gene duplicates being maintained to increase gene dosage/expression levels [31–33]. In addition, gene duplication has also been shown to contribute to the robustness of complex formation [68]. We also found that dynamically changing paralogs can be anti-correlated. Here we showed that anti-correlated paralogous pairs have a greater amino acid sequence and Pfam domain divergence and are evolutionary older genes, suggesting that paralogs have attained specific roles during differentiation.

To analyze how dynamic and non-dynamic genes are integrated into complexes, we used the CORUM database to map the changes in gene expression onto protein complexes. Earlier work analyzing complex assemblies during the yeast cell cycle revealed that complexes consist of both constitutively non-dynamic and dynamically expressed subunits (‘just-in-time assembly’) [6]. Our study of a mammalian differentiation process augments these efforts as we find a larger fraction of di-chromatic complexes, containing both dynamic and non-dynamic genes. When we applied these concepts to a manually annotated large signaling complex to include directionality, the TNF-α/NF-κB signaling, we found a stable core associated to both a dynamically changing module and several stable modules. We propose this as a ‘constant signalosome ready to work’, where a stable core—involving TNF receptor/TRAF2—associates with a dynamic periphery with different functions: i.e. NF-κB signaling, TRAF1 resulting in anti-apoptotic effects without affecting TRAF2/TRAD. On the level of EGFR signaling, a picture emerges whereby several sub-complexes consisting of adaptors, Raf kinases, ERK kinases, etc. show a mix of both, dynamic and non-dynamic subunits. In contrast to what is proposed in the hour-glass model of signaling [69,70], our analysis suggests that several sub-complexes act in parallel, containing both common non-dynamic and different dynamic subunits.

An alternative view of complexes (‘machines vs. ensembles’) has been proposed by Loew and colleagues, which holds that in addition to stable signaling complexes and molecular machines, such as the ribosome or the proteasome, a considerable combinatorial complexity arises from different compositions of complexes [8,71,72]. We propose that these two concepts do not exclude each other and are probably both used in signal transduction. However, it is currently unclear how ‘ensemble-like’ signaling complexes actually are. Both, computational and experimental work is critical to answer this question and in particular structural analyses help to identify the two cases by distinguishing compatible (more machine-like) from mutually exclusive (more ensemble-like) complexes [10,71].

Structural information about protein domains was used to distinguish compatible and mutually exclusive protein-protein interactions among hub proteins and their binding partners [10,19]. Here, we used the SAPIN webserver to distinguish compatible from mutually exclusive binding surfaces [19]. Other successful methods for structural characterization are based on evolutionary conservation of the interacting residues, e.g. PrePPI [73], Inferred Biomolecular Interaction Server—IBIS webserver [74], and Interactome3D [15]. We identified that dynamic genes binding to a common non-dynamic hub are enriched in mutually exclusive interactions (XOR), suggesting that changes in complex assemblies during differentiation could also be caused through variation in the abundance of proteins that compete for binding to XOR nodes [14]. In fact we show here that XOR nodes involving super-dynamic genes are often from different clusters, supporting this hypothesis. Thus we go one step further and propose a model where at least some protein complexes exist assembled stably at the core, and their behavior is modified by the attachment and detachment of accessory proteins at the periphery of the signaling cascade in response to various conditions. This is in agreement with the concept of achieving cell type-specific complexes through the interaction of core (cell/tissue general) and peripheral (cell type/tissue-specific) proteins [7,75]. However, more detailed computational modeling, including information on XOR and AND nodes are needed to investigate the global impact on protein competition, e.g. how changes in protein abundance propagate through a larger PPI network [71].

We have found examples where anti-correlated changes in gene expression of subunits in a complex (Ubiqutin/degradation), or in two complexes competing for the same component (EGFR recycling complexes), which reflect very nicely what is known about factors important for keratinocyte differentiation. This supports the idea of protein competition as a driver of biological processes. In fact, computational modeling of the yeast PPI network has suggested that changes in concentration spread locally and decrease exponentially within the network, as a function of the distance from the perturbed node [76]. Previous experimental and computational work investigating competition at the RAS node has confirmed these rather local effects of competition [14]. Furthermore, it is known that competing protein interactions can induce switch-like cellular behaviors, such as apoptosis versus differentiation [77], or self-renewal versus differentiation [78]. However, cellular fates also crucially depend on the crosstalk between signaling pathways initiated at different phosphorylation sites providing spatiotemporal separation and acting as molecular switches [79]. As our gene expression changes did not allow us to analyze phosphorylation events, it is difficult to speculate to what extend phosphorylation levels contribute to network and complex reorganization processes during keratinocyte differentiation. Likewise, the role of homooligomeric complexes is neglected despite being the dominating type of interaction. In the future, it will be important to integrate protein interaction networks resolved at the level of domains as well as phosphorylation events and homooligomeric complexes, in order to provide the complete picture.

Altogether our analysis highlights the importance of understanding the dynamic assembly and disassembly of complexes taking 3D structural information into consideration, rather than unraveling networks of individual proteins.

## Materials and Methods

### Microarrays, data normalization and filtering

The time-course microarray data analyzed in this work was generated in our previous study [3]. However, in this section of the methods, we re-outline the details of this experiment. Total RNA in the amount of 500 ng from calcium treated primary human keratinocytes collected every 5 hours in triplicates for a total of 45 hours (i.e. 10 samples) and labelled using Agilent LowInputQuick Amp Labelling kit following manufacturer instructions. mRNA was reverse transcribed in the presence of T7-oligo-dT primer to produce cDNA, The cDNA was then in vitro transcribed with T7 RNA polymerase in the presence of Cy3-CTP to produce labeled cRNA and this labeled cRNA was in turn hybridized to Agilent Human Gene Expression 4x44K v2 microarrays (ID026652). Signals from probes were obtained by the Agilent’s Feature Extraction custom software, were corrected for background noise using the normexp method [80] available in the R package limma from Bioconductor, and then normalized between arrays to assure comparability across samples using quantile normalization [81]. Lastly, the dataset was log2-transformed. The dataset was filtered to remove and collapse all Agilent control spots and to only include probes that were present above stochastic background expression. To do this we filtered any probes with expression profiles completely below 7 which is a figure obtained by taking the median of the entire dataset and rounding it down to a single digit. This procedure yielded 21,113 probes, which were then mapped to 16,720 genes where the probe with the highest mean expression represents a gene. For some analyses such as K-means clustering the dataset was mean-centered and scaled such that the mean of each gene is zero.

### Classification into constitutive and dynamic genes

To distinguish genes that are changing (i.e. transiently or continuously up- or down-regulated) from those that are constant (i.e. stably expressed) across our differentiation time-course, we used Chi-squared test where for each gene, the expected value at each time point is equivalent to the gene’s average expression across all 10. The equation is as follow:

$${\text{X}}^{2}=\frac{\sum_{\text{i}}^{\text{N}}{\left({\bar{\text{X}}}_{\text{i}}-\bar{\text{X}}\right)}^{2}}{\text{N}-1}$$

where N is the number of samples or time points (i.e. 10), ${\bar{\text{X}}}_{\text{i}}$ is the mean expression of gene x across three experimental repeats at time point i, and $\bar{\text{X}}$ is the overall mean expression of gene x across all 10 time points and all repeats. Note that in this formula there is no normalization of ${\left({\bar{\text{X}}}_{\text{i}}-\bar{\text{X}}\right)}^{2}$ by the experimental error computed as $\text{SE = SD}/\sqrt{\text{N}}$. Instead we computed a universal error and scaled the X^2^ distributions of our data to the standard X^2^ distribution for 9 degrees of freedom.

In addition to Chi-squared test we employed an empirically derived fold change threshold of 2 and a strict threshold of 4. Each gene’s expression peak was compared to its trough to ensure the difference satisfies the appropriate threshold and the genes were classified as follow:

1. non-dynamic genes have to satisfy P(X^2^)> = 0.01 and fold change < 2
2. unresolved genes either have P(X^2^)<0.01 and fold change < = 2 or P(X^2^) > = 0.01 and fold change > = 2
3. dynamic genes P(X^2^)<0.01 and fold change> = 2.
4. super-dynamic genes are a subset of dynamic genes which have fold change> = 4

We also conducted corrections for multiple testing using Benjamini’s method. While the above analysis had classified 6,096 genes as dynamic (1,317 as super-dynamic), after Benjamini correction for multiple testing and applying a p-value cutoff of 0.01 (1%), these two numbers remained the same while the number of non-dynamic genes increased from 6,137 to 6,413 and the number of unresolved genes decreased from 4,487 to 4,211. Thus, a subset of 200 genes got shifted from the unresolved category to the non-dynamic category after Benjamini correction. Since the majority of our analyses were based on dynamic and super-dynamic genes such as clustering and mapping to complexes and these classes did not change after multiple testing correction, we kept the gene classes as originally defined without Benjamini correction.

### Statistical and bioinformatics analyses

We used R version 2.14 for the majority of the statistical analyses along with Perl and AWK for text processing. K-means clustering was performed using the R kmeans function. To optimize and select the K value or the number of cluster centers, we exhaustively performed K-means clustering with K value ranging from 2 to 500 and then for each set of clusters, computed an F-score as well as an average silhouette score available from the standard statistics and cluster packages, respectively. The K value, which resulted in the highest average silhouette score and F-score, was selected as the optimal number of cluster centers.

### Protein complex analysis

A reference set of complexes was obtained from the CORUM database [36] of curated mammalian protein complexes. Out of a total of 1,331 human complexes available in the latest release (February 17^th^, 2012), 44 homo-oligomer complexes consisting of only one type of protein were filtered out. 84 complexes were removed, which were fully non-expressed across our skin differentiation expression time course. 154 (<13%) complexes contain subunits that are partially non-expressed in keratinocytes while 1,049 complexes contain subunits, which are all expressed during skin differentiation. The resulting set of 1,203 complexes consists of 2 or more distinct proteins per complex.

### Paralogous gene annotations

Paralog annotations were obtained from EnsemblCompara through the BioMart portal (database version EnsemblGenes71). The method identifies true paralogs by computing a phylogenetic tree across the whole set of protein-coding genes with one pipeline which includes TreeBeST [33].

### Structural analysis

Analysis of mutually exclusive (XOR) and compatible (AND) binding was done using the SAPIN web framework [19]. SAPIN identifies the protein parts that could be involved in the interaction and provides template structures and then performs structural superimpositions to identify compatible and mutually exclusive interactions. We analyzed all complexes in the CORUM database with a complex size of less than 20 members. Each CORUM complex was broken down into combinations of three different proteins, and the respective protein sequences were used as the input for the SAPIN webserver. The workflow in SAPIN is:

1. Assigning all domains using Pfam (pfam_scan.pl with default parameter) based on the protein sequences.
2. Searching the 3DID [82] database to find potential domain-domain interaction hits of binary interaction partners provided.
3. Finding the best template of PPI structures (containing interacting domain A and domain B) by using the InterPreTS scoring function [83]. The interaction structures are evaluated by InterPreTS based on interface sequences aligned with 3Dstructures (we used MUSCLE [84] with default parameter). SAPIN selects one template that has more than 2.33 Z-score or the best score one among all candidates.
4. If two PPI structures share a same domain, structural superimpositions are performed based on the domain structure using Combinatorial Extension (CE [85]).
5. Analyzing for backbone clashes using FoldX [86] with superposed structures, and based on a backbone clashes threshold (15% of interface residues) the interactions are assigned compatible (AND) or mutually exclusive (XOR).
6. The reliability of the predictions with respect to sequence similarity to the template complex, we measured the sequence identity between the reference and homolog domains from the alignment based on the Needleman-Wunsch Algorithm [87]. Based on this, we determined a Z-score for the percentage of van der Waals backbone clashing among interface residues calculated for either AND or XOR, dependent on sequence similarity [19].

If a protein has at least two interacting partners, the domains mediating the interaction are superimposed on the reference domain, and the interacting domains are analyzed for compatibility (AND) or mutual exclusiveness (XOR). Note, that even two protein having similar domains do not necessarily bind at the same site/interface, but there are frequent cases of similar domains binding in a different way. As the best template suggestion based on sequence homology using INTERPRETS [9]. As the sequence is scored in INTERPRETS against a set of 1000 random sequences, the selection of the best template is not deterministic and can result in different results in different runs. We provide the average of the XOR/AND likelihood of independent runs in S4 Table.

### Cell culture and differentiation of primary keratinocytes

For calcium-induced differentiation experiments, keratinocytes were seeded into 35 mm plates and grown in Keratinocyte Serum-Free Medium with supplements (KSFM; GIBCO) [3]. After reaching 70% confluence Keratinocyte Serum-Free Medium was exchanged for EMEM (Lonza) supplemented with 8% chelated FBS, EGF (10 ng/ml), 1% penicillin/streptomycin and 0.05 mM CaCl2. After 12 hour time point 0 h was collected, and the residual keratinocytes were synchronized for 2 h with EMEM containing 20% chelated FBS, EGF (10 ng/ml), 1% penicillin/streptomycin, and 0.05 mM CaCl2. After synchronization cells were washed once with PBS and cultured in EMEM, supplemented with 8% chelated FBS, EGF (10 ng/ml), 1% penicillin/streptomycin, and either 0.05 mM or 1.2 mM CaCl2, corresponding to non-calcium and calcium treatment, respectively. Cells were collected every 5 hours during a period of 45 hr.

### Immunostaining and imaging

Keratinocytes were seeded onto 16-chambered LabTek slides (Nuncbrand). Kerationocyte differentiation was induced through addition of Calcium (as before), and sample were taken at the respective time points. Cells were fixed in 4% paraformaldehyde for 20 min and blocked with 4% BSA in PBS 1× (blocking solution). Primary staining was done using an antibody against Involucrin (Abcam, ab28057; dilution 1:1000 in blocking solution) followed by an Alexa Fluor 568 secondary antibody (Invitrogen Probes; dilution 1:200 in blocking solution). Cells were counterstained with DAPI (Sigma-Aldrich; concentration 1 μg/mL) and mounted using Mowiol solution. Staining was visualized on a Leica TCS SP5 confocal microscope with a 40× 1.25 NA objective at a zoom factor of 3 (1024 × 1024 pixels; 0.126 μm/pixel).

## Supporting Information

S1 Fig Functional analysis of 1317 super-dynamic proteins. Classification was based on DAVID, UniProt, and manual literature searches into proteins needed for the formation of the cornified envelope (‘Keratinocyte Differentiation’), Metabolism, general signaling, and housekeeping. For some proteins only a Pfam domain prediction can be assigned (‘Domain Info’), or have no domain and an unknown function (‘unknown Function). The remaining proteins are not annotated (‘unknown Gene Protein’).
(TIF)

S2 Fig Functional analysis of 417 super-dynamic signaling-related proteins. Further functional sub-classification of the proteins related to signaling based on DAVID, UniProt, and manual literature searches.
(TIF)

S3 Fig Functional analysis of 292 super-dynamic housekeeping-related proteins. Further functional sub-classification of the housekeeping proteins based on DAVID, UniProt, and manual literature searches.
(TIF)

S4 Fig Functional analysis of 172 super-dynamic metabolism-related proteins. Further functional sub-classification of the proteins related to metabolism based on DAVID, UniProt, and manual literature searches.
(TIF)

S5 Fig Functional analysis of 104 super-dynamic proteins related to keratinocyte function and differentiation. Further functional sub-classification of the proteins related to keratinocyte differentiation (‘Keratinocyte Differentiation’) based on DAVID, UniProt, and manual literature searches.
(TIF)

S6 Fig Involucrin as a marker of keratinocyte differentiation confirmed by immunofluorescence. Comparing gene expression changes (bar diagrams) with protein expression of involucrin (IVL) by immunostaining (inserted pictures) at different time points after calcium treatment.
(TIF)

S7 Fig Statistics for correlated and anti-correlated super-dynamic and dynamic proteins. Density plots of Pearson correlation coefficients (PCC) of gene expression comparison of (A) paralog super-dynamic gene pairs (n = 340, mean = 0.6, median = 0.8) to random super-dynamic gene pairs (n = 340, mean = 0, median = 0) indicates a real shift in distributions (P = 4.5e−37) and also (B) paralog dynamic gene pairs (n = 2,260, mean = 0.3, median = 0.4) to random dynamic gene pairs (n = 2,260, mean = 0, median = 0) indicates a real shift in distributions (P = 5.4e−61). Statistical significance tested using Wilcoxon rank sum test.
(TIF)

S8 Fig Examples for super-dynamic correlated paralog pairs grouped into similar paralog families. The expression plotted compared to time 0 against time after calcium induction (log transformed data).
(TIF)

S9 Fig Examples for super-dynamic anti-correlated paralog pairs grouped into similar paralog families. The expression plotted compared to time 0 against time after calcium induction (log transformed data).
(TIF)

S10 Fig Comparison of difference in the number of Pfam domains among of correlated and anti-correlated dynamic paralogous protein pairs. Dynamic anti-correlated paralogs (n = 281, r< = −0.6) have a mean of 1.7 differences in the total number of domains compared to dynamic correlated paralogs (n = 953, r> = 0.6) which have a mean of 1.3 total differences in how many Pfam domains they have (P = 0.008).
(TIF)

S11 Fig Comparison of sequence features of correlated and anti-correlated paralogous protein pairs. (A) Dynamic anti-correlated paralogs (n = 281, r< = -0.6) have on average 34% sequence homology compared to dynamic correlated paralogs (n = 949, r> = 0.6) which are on average 46% similar at sequence level (P = 3.8e−11). (B) Super-dynamic anti-correlated paralogs (n = 19, r< = −0.6) have on average 34% sequence homology compared to 51% for super-dynamic correlated paralogs (n = 234, r> = 0.6) (P = 0.005). (C) Dynamic anti−correlated paralog pairs (n = 281, r< = −0.6) have an average 330 residue-difference in their sequence length compared to correlated paralog pairs (n = 952,r> = 0.6) which have an average sequence length difference of 227 residues (P = 8.3e−08). (D) Super-dynamic anti−correlated paralog pairs (n = 19, r< = −0.6) have a mean sequence length difference of 247 residues compared to correlated paralogs (n = 235, r> = 0.6) which have a mean difference of 128 (P = 0.01). Statistical significance tested using Wilcoxon rank sum test.
(TIF)

S12 Fig Comparison of duplication age of correlated and anti-correlated paralogous protein pairs. (A) comparison of dynamic anti-correlated paralog pairs (n = 281, r< = -0.6) to dynamic correlated paralog pairs (n = 953, r> = 0.6) indicates that 90% of the former group are the result of old duplication events compared to 58% of the latter (P = 1.5e−25), 10% are the result of intermediate duplication events compared to 30% (P = 4. 5e−13) and finally 1% are from recent duplication events compared to 12% (1.4e−11). (B) comparison of super-dynamic anti-correlated paralog pairs (n = 19, r< = -0.6) to super-dynamic correlated paralog pairs (n = 235, r> = 0.6) indicates that 90% of the former group are the result of old duplication events compared to 27% of the latter (P = 1.05e−07), 11% are the result of intermediate duplication events compared to 48% (P = 0.001), and lastly 0% are from recent duplication events compared to 24% (P = 0.009). Statistical significance tested using Fisher's exact test.
(TIF)

S13 Fig Global map of human protein complexes classified according to the dynamic expression change. Protein expression changes mapped on the human complexes from CORUM. The node color represents the expression category (see legend). Same pattern observed by others before emerges in the global map whereby complexes are usually a mix of both non-dynamically and dynamically expressed genes. About 20% of protein complexes showed statistically significant (p. < 0.05) co-regulation in the expression profiles of their subunits. Similarly a large number of protein families and pathways showed concerted temporal dynamics among their components (S3 Table). Complexes are highly inter-connected.
(TIF)

S14 Fig Selection of dichromatic complexes. Selected CORUM complexes of similar core proteins were plotted. Super-dynamic and dynamic genes were colored according to clusters. Black indicates constitutive genes, grey indicated unresolved genes.
(TIF)

S15 Fig Manual literature assembled TNF-α/NF-κB receptor signaling complex network and mapping of gene expression changes. The manually-curated network is based on [44] and [45]. Genes are colored based on expression classification (see legend). TNFRSF1A is the TNF receptor with broad tissue expression. Other TNF receptor and TRAF family members are displayed in boxes on the left side. See legend for details.
(TIF)

S16 Fig Manual literature assembled ErbB receptor signaling complex network and mapping of gene expression changes. Genes are colored based on expression classification (see legend).
(TIF)

S17 Fig Protein complex sizes in CORUM and selecting a threshold for complexes analyzed by SAPIN. (A) Distribution of protein complex sizes (number of subunits) indicates that the majority of complexes have less than 20 subunits. (B) We impose a threshold of 20 on complex size for the downstream SAPIN-mediated structural analyses (S4 Table).
(TIF)

S18 Fig Network representation of combining expression classification with compatible (AND) and mutually exclusive (XOR) interaction types. Network representation of compatibility scores calculated using SAPIN. We show only non-dynamic and dynamic cases and not unresolved cases. The edge color and width corresponds to the average surface compatibility. The nodes are Nodes colored according to expression or and cluster membership. Please see the legend for details. The network is represented using Cytoscape.
(TIF)

S19 Fig Schematic representation of possible cases combining expression classification with different surface interaction types: compatible interactions (AND) and mutually exclusive interactions (XOR). There are a total of 18 possible cases if we take all expression classes—dynamic/super-dynamic, non-dynamic, unresolved—into account.
(TIF)

S20 Fig Case counts involving expression classes: dynamic, non-dynamic, and unresolved (refer to S19 Fig for case descriptions). (A) Frequency (count data) comparison of AND and XOR for 18 cases. (B) Percentage (ratios) comparison of AND and XOR for 18 cases.
(TIF)

S21 Fig Case counts involving expression classes: super-dynamic, non-dynamic, and unresolved (refer to S19 Fig for case descriptions). (A) Frequency (count data) comparison of AND and XOR for 18 cases. (B) Percentage (ratios) comparison of AND and XOR for 18 cases.
(TIF)

S1 Table Gene expression changes during calcium-induced differentiation of human primary keratinocyte stem cells and GO analysis. (XLSX)

S2 Table Paralogous pairs constitutively or dynamically changing during keratinocyte differentiation. (XLSX)

S3 Table Gene expressed changes mapped on CORUM protein complexes. (XLSX)

S4 Table Structural analysis of CORUM complexes using the SAPIN framework. (XLSX)

S1 Network Network representation in Fig 4 as cytoscape file. (ZIP)

## References

[1] Prudhomme W., Daley G. Q., Zandstra P., Lauffenburger D. A., Multivariate proteomic analysis of murine embryonic stem cell self-renewal versus differentiation signaling. *Proc*. *Natl*. *Acad*. *Sci*. *U*.*S*.*A*. 101, 2900–2905 (2004). 14978270 10.1073/pnas.0308768101 PMC365717

[2] Novershtern N., Subramanian A., Lawton L. N., Mak R. H., Haining W. N., McConkey M. E., Habib N., Yosef N., Chang C. Y., Shay T., Frampton G. M., Drake A. C., Leskov I., Nilsson B., Preffer F., Dombkowski D., Evans J. W., Liefeld T., Smutko J. S., Chen J., Friedman N., Young R. A., Golub T. R., Regev A., Ebert B. L., Densely interconnected transcriptional circuits control cell states in human hematopoiesis. *Cell* 144, 296–309 (2011). 10.1016/j.cell.2011.01.004 21241896 PMC3049864

[3] Janich P., Toufighi K., Solanas G., Luis N. M., Minkwitz S., Serrano L., Lehner B., Benitah S. A., Human Epidermal Stem Cell Function Is Regulated by Circadian Oscillations. *Cell Stem Cell* 13, 745–753 (2013). 10.1016/j.stem.2013.09.004 24120744

[4] Hansson J., Rafiee M. R., Reiland S., Polo J. M., Gehring J., Okawa S., Huber W., Hochedlinger K., Krijgsveld J., Highly coordinated proteome dynamics during reprogramming of somatic cells to pluripotency. *Cell Rep*. 2, 1579–1592 (2012). 10.1016/j.celrep.2012.10.014 23260666 PMC4438680

[5] von Kriegsheim A., Baiocchi D., Birtwistle M., Sumpton D., Bienvenut W., Morrice N., Yamada K., Lamond A., Kalna G., Orton R., Gilbert D., Kolch W., Cell fate decisions are specified by the dynamic ERK interactome. *Nat*. *Cell Biol*. 11, 1458–1464 (2009). 10.1038/ncb1994 19935650 PMC3839079

[6] de Lichtenberg U., Jensen L. J., Brunak S., Bork P., Dynamic complex formation during the yeast cell cycle. *Science* 307, 724–727 (2005). 15692050 10.1126/science.1105103

[7] Bossi A., Lehner B., Tissue specificity and the human protein interaction network. *Mol*. *Sys*. *Biol*. 5, 260 (2009). 10.1038/msb.2009.17 PMC2683721 19357639

[8] Mayer B. J., Blinov M. L., Loew L. M., Molecular machines or pleiomorphic ensembles: signaling complexes revisited. *J*. *Biol*. 8, 81 (2009). 19835637 10.1186/jbiol185 PMC2776906

[9] Aloy P., Russell R. B., Interrogating protein interaction networks through structural biology. *Proc*. *Natl*. *Acad*. *Sci*. *U*.*S*.*A*. 99, 5896–5901 (2002). 11972061 10.1073/pnas.092147999 PMC122873

[10] Kim P. M., Lu L. J., Xia Y., Gerstein M. B., Relating three-dimensional structures to protein networks provides evolutionary insights. *Science* 314, 1938–1941 (2006). 17185604 10.1126/science.1136174

[11] Kiel C., Beltrao P., Serrano L., Analyzing protein interaction networks using structural information. *Annu*. *Rev*. *Biochem*. 77, 415–441 (2008). 10.1146/annurev.biochem.77.062706.133317 18304007

[12] Stein A., Mosca R., Aloy P., Three-dimensional modeling of protein interactions and complexes is going 'omics. *Curr*. *Opin*. *Struct*. *Biol*. 21, 200–208 (2011). 10.1016/j.sbi.2011.01.005 21320770

[13] Kiel C., Vogt A., Campagna A., Chatr-aryamontri A., Swiatek-de Lange M., Beer M., Bolz S., Mack A. F., Kinkl N., Cesareni G., Serrano L., Ueffing M., Structural and functional protein network analyses predict novel signaling functions for rhodopsin. *Mol*. *Syst*. *Biol*. 7, 551 (2011). 10.1038/msb.2011.83 22108793 PMC3261702

[14] Kiel C., Verschueren E., Yang J. S., Serrano L., Integration of Protein Abundance and Structure Data Reveals Competition in the ErbB Signaling Network. *Sci*. *Signal*. 6, ra109 (2013). 10.1126/scisignal.2004560 24345680

[15] Mosca R., Ceol A., Aloy P., Interactome3D: adding structural details to protein networks. *Nat*. *Methods* 10, 47–53 (2013). 23399932 10.1038/nmeth.2289

[16] Dotto G. P., Signal transduction pathways controlling the switch between keratinocyte growth and differentiation. *Crit*. *Rev*. *Oral Biol*. *Med*. 10, 442–457 (1999). 10634582 10.1177/10454411990100040201

[17] Cheng X., Jin J., Hu L., Shen D., Dong X. P., Samie M. A., Knoff J., Eisinger B., Liu M. L., Huang S. M., Caterina M. J., Dempsey P., Michael L. E., Dlugosz A. A., Andrews N. C., Clapham D. E., Xu H., TRP channel regulates EGFR signaling in hair morphogenesis and skin barrier formation. *Cell* 141, 331–343 (2010). 20403327 10.1016/j.cell.2010.03.013 PMC2858065

[18] Hennings H., Michael D., Cheng C., Steinert P., Holbrook K., Yuspa S. H., Calcium regulation of growth and differentiation of mouse epidermal cells in culture. *Cell* 19, 245–254 (1980). 6153576 10.1016/0092-8674(80)90406-7

[19] Yang J. S., Campagna A., Delgado J., Vanhee P., Serrano L., Kiel C., SAPIN: a framework for the structural analysis of protein interaction networks. *Bioinformatics* 28, 2998–2999 (2012). 10.1093/bioinformatics/bts539 22954630

[20] Huang D. W., Sherman B. T., Tan Q., Kir J., Liu D., Bryant D., Guo Y., Stephens R., Baseler M. W., Lane H. C., Lempicki R. A., DAVID Bioinformatics Resources: expanded annotation database and novel algorithms to better extract biology from large gene lists. *Nucleic Acids Res*. 35, W169–175 (2007). 17576678 10.1093/nar/gkm415 PMC1933169

[21] Huang D. W., Sherman B. T., Lempicki R. A., Systematic and integrative analysis of large gene lists using DAVID bioinformatics resources. *Nat*. *Protoc*. 4, 44–57 (2009). 10.1038/nprot.2008.211 19131956

[22] Magrane M., Consortium U., UniProt Knowledgebase: a hub of integrated protein data. *Database*, bar009 (2011). 10.1093/database/bar009 21447597 PMC3070428

[23] Candi E., Schmidt R., Melino G., The cornified envelope: a model of cell death in the skin. *Nat*. *Rev*. *Mol*. *Cell*. *Biol*. 6, 328–340 (2005). 15803139 10.1038/nrm1619

[24] Andreoli J. M., Jang S. I., Chung E., Coticchia C. M., Steinert P. M., Markova N. G., The expression of a novel, epithelium-specific ets transcription factor is restricted to the most differentiated layers in the epidermis. *Nucleic Acids Res*. 25, 4287–4295 (1997). 9336459 10.1093/nar/25.21.4287 PMC147045

[25] Oettgen P., Alani R. M., Barcinski M. A., Brown L., Akbarali Y., Boltax J., Kunsch C., Munger K., Libermann T. A., Isolation and characterization of a novel epithelium-specific transcription factor, ESE-1, a member of the ets family. *Mol Cell Biol*. 17, 4419–4433 (1997). 9234700 10.1128/mcb.17.8.4419 PMC232296

[26] Sementchenko V. I., Watson D. K., Ets target genes: past, present and future. *Oncogene* 19, 6533–6548 (2000). 11175369 10.1038/sj.onc.1204034

[27] Brembeck F. H., Opitz O. G., Libermann T. A., Rustgi A. K., Dual function of the epithelial specific ets transcription factor, ELF3, in modulating differentiation. *Oncogene* 19, 1941–1949 (2000). 10773884 10.1038/sj.onc.1203441

[28] Kolly C., Suter M. M., Muller E. J., Proliferation, cell cycle exit, and onset of terminal differentiation in cultured keratinocytes: pre-programmed pathways in control of C-Myc and Notch1 prevail over extracellular calcium signals. *J*. *Invest*. *Dermatol*. 124, 1014–1025 (2005). 15854044 10.1111/j.0022-202X.2005.23655.x

[29] Brissette J. L., Kumar N. M., Gilula N. B., Hall J. E., Dotto G. P., Switch in gap junction protein expression is associated with selective changes in junctional permeability during keratinocyte differentiation. *Proc*. *Natl*. *Acad*. *Sci*. *U*.*S*.*A*. 91, 6453–6457 (1994). 8022804 10.1073/pnas.91.14.6453 PMC44220

[30] Vilella A. J., Severin J., Ureta-Vidal A., Heng L., Durbin R., Birney E., EnsemblCompara GeneTrees: Complete, duplication-aware phylogenetic trees in vertebrates. *Genome Res*. 19, 327–335 (2009). 10.1101/gr.073585.107 19029536 PMC2652215

[31] Kondrashov F. A., Koonin E. V., A common framework for understanding the origin of genetic dominance and evolutionary fates of gene duplications. *Trends Genet*. 20, 287–290 (2004). 15219392 10.1016/j.tig.2004.05.001

[32] Perry G. H., Dominy N. J., Claw K. G., Lee A. S., Fiegler H., Redon R., Werner J., Villanea F. A., Mountain J. L., Misra R., Carter N. P., Lee C., Stone A. C., Diet and the evolution of human amylase gene copy number variation. *Nat*. *Genet*. 39, 1256–1260 (2007). 17828263 10.1038/ng2123 PMC2377015

[33] Qian W., Zhang J., Gene dosage and gene duplicability. *Genetics* 179, 2319–2324 (2008). 10.1534/genetics.108.090936 18689880 PMC2516101

[34] Pulimeno P., Paschoud S., Citi S., A role for ZO-1 and PLEKHA7 in recruiting paracingulin to tight and adherens junctions of epithelial cells. *J*. *Biol*. *Chem*. 286, 16743–16750 (2011). 10.1074/jbc.M111.230862 21454477 PMC3089516

[35] Shen L., Qu X., Ma Y., Zheng J., Chu D., Liu B., Li X., Wang M., Xu C., Liu N., Yao L., Zhang J., Tumor suppressor NDRG2 tips the balance of oncogenic TGF-beta via EMT inhibition in colorectal cancer. *Oncogenesis* 3, e86 (2014). 10.1038/oncsis.2013.48 24492480 PMC3940918

[36] Ruepp A., Waegele B., Lechner M., Brauner B., Dunger-Kaltenbach I., Fobo G., Frishman G., Montrone C., Mewes H. W., CORUM: the comprehensive resource of mammalian protein complexes—2009. *Nucleic Acids Res*. 38, D497–501 (2010). 10.1093/nar/gkp914 19884131 PMC2808912

[37] Sääf A., Pivarcsi A., Winge M.C., Wahlgren C.F., Homey B., Nordenskjöld M., Tengvall-Linder M., Bradley M., Characterization of EGFR and ErbB2 expression in atopic dermatitis patients. *Arch*. *Dermatol*. *Res*. 304, 773–780 (2012). 10.1007/s00403-012-1242-4 22552355

[38] Kong C., Su X., Chen P. I., Stahl P. D., Rin1 interacts with signal-transducing adaptor molecule (STAM) and mediates epidermal growth factor receptor trafficking and degradation. *J*. *Biol*. *Chem*. 282, 15294–15301 (2007). 17403676 10.1074/jbc.M611538200

[39] Feng L., Wang J. T., Jin H., Qian K., Geng J. G., SH3KBP1-binding protein 1 prevents epidermal growth factor receptor degradation by the interruption of c-Cbl-CIN85 complex. *Cell Biochem*. *Funct*. 29, 589–596 (2011). 10.1002/cbf.1792 21830225 PMC4534006

[40] Bertelsen V., Stang E., The Mysterious Ways of ErbB2/HER2 Trafficking. *Membranes* 4, 424–446 (2014). 25102001 10.3390/membranes4030424 PMC4194043

[41] van Hogerlinden M., Rozell B. L., Ahrlund-Richter L., Toftgard R., Squamous cell carcinomas and increased apoptosis in skin with inhibited Rel/nuclear factor-kappaB signaling. *Cancer Res*. 59, 3299–3303 (1999). 10416581

[42] Lippens S., Lefebvre S., Gilbert B., Sze M., Devos M., Verhelst K., Vereecke L., Mc Guire C., Guerin C., Vandenabeele P., Pasparakis M., Mikkola M. L., Beyaert R., Declercq W., van Loo G., Keratinocyte-specific ablation of the NF-kappaB regulatory protein A20 (TNFAIP3) reveals a role in the control of epidermal homeostasis. *Cell Death Differ*. 18, 1845–1853 (2011). 21566665 10.1038/cdd.2011.55 PMC3214908

[43] Banno T., Gazel A., Blumenberg M., Effects of tumor necrosis factor-alpha (TNF alpha) in epidermal keratinocytes revealed using global transcriptional profiling. *J*. *Biol*. *Chem*. 279, 32633–32642 (2004). 15145954 10.1074/jbc.M400642200

[44] Bouwmeester T., Bauch A., Ruffner H., Angrand P. O., Bergamini G., Croughton K., Cruciat C., Eberhard D., Gagneur J., Ghidelli S., Hopf C., Huhse B., Mangano R., Michon A. M., Schirle M., Schlegl J., Schwab M., Stein M. A., Bauer A., Casari G., Drewes G., Gavin A. C., Jackson D. B., Joberty G., Neubauer G., Rick J., Kuster B., Superti-Furga G., A physical and functional map of the human TNF-alpha/NF-kappa B signal transduction pathway. *Nat*. *Cell Biol*. 6, 97–105 (2004). 14743216 10.1038/ncb1086

[45] Napetschnig J., Wu H., Molecular basis of NF-kappaB signaling. *Annu*. *Rev*. *Biophys*. 42, 443–468 (2013). 10.1146/annurev-biophys-083012-130338 23495970 PMC3678348

[46] Chen G., Goeddel D. V., TNF-R1 signaling: a beautiful pathway. *Science* 296, 1634–1635 (2002). 12040173 10.1126/science.1071924

[47] Dajee M., Lazarov M., Zhang J. Y., Cai T., Green C. L., Russell A. J., Marinkovich M. P., Tao S., Lin Q., Kubo Y., Khavari P. A., NF-kappaB blockade and oncogenic Ras trigger invasive human epidermal neoplasia. *Nature* 421, 639–643 (2003). 12571598 10.1038/nature01283

[48] Oeckinghaus A., Hayden M. S., Ghosh S., Crosstalk in NF-kappaB signaling pathways. *Nat Immunol*. 12, 695–708 (2011). 10.1038/ni.2065 21772278

[49] Lee S. Y., Choi Y., TRAF1 and its biological functions. *Adv*. *Exp*. *Med*. *Biol*. 597, 25–31 (2007). 17633014 10.1007/978-0-387-70630-6_2

[50] Xie P., TRAF molecules in cell signaling and in human diseases. *J*. *Mol*. *Signal*. 8, 7 (2013). 23758787 10.1186/1750-2187-8-7 PMC3697994

[51] Fotin-Mleczek M., Henkler F., Hausser A., Glauner H., Samel D., Graness A., Scheurich P., Mauri D., Wajant H., Tumor necrosis factor receptor-associated factor (TRAF) 1 regulates CD40-induced TRAF2-mediated NF-kappaB activation. *J*. *Biol*. *Chem*. 279, 677–685 (2004). 14557256 10.1074/jbc.M310969200

[52] Irmler M., Steiner V., Ruegg C., Wajant H., Tschopp J., Caspase-induced inactivation of the anti-apoptotic TRAF1 during Fas ligand-mediated apoptosis. *FEBS Lett*. 468, 129–133 (2000). 10692572 10.1016/s0014-5793(00)01206-0

[53] Zhang L. J., Bhattacharya S., Leid M., Ganguli-Indra G., Indra A. K., Ctip2 is a dynamic regulator of epidermal proliferation and differentiation by integrating EGFR and Notch signaling. *J*. *Cell Sci*. 125, 5733–5744 (2012). 10.1242/jcs.108969 23015591 PMC3575708

[54] Sarikas A., Hartmann T., Pan Z. Q., The cullin protein family. *Genome Biol*. 12, 220 (2011). 10.1186/gb-2011-12-4-220 21554755 PMC3218854

[55] Bornstein G., Ganoth D., Hershko A., Regulation of neddylation and deneddylation of cullin1 in SCFSkp2 ubiquitin ligase by F-box protein and substrate. *Proc*. *Natl*. *Acad*. *Sci*. *U*. *S*. *A*. 103, 11515–11520 (2006) 16861300 10.1073/pnas.0603921103 PMC1544201

[56] McDade S. S., Patel D., McCance D. J., p63 maintains keratinocyte proliferative capacity through regulation of Skp2-p130 levels. *J*. *Cell Sci*. 124, 1635–1643 (2011). 10.1242/jcs.084723 21511729 PMC3085435

[57] Dubiel D., Gierisch M. E., Huang X., Dubiel W., Naumann M., CAND1-dependent control of cullin 1-RING Ub ligases is essential for adipogenesis. *Biochim*. *Biophys*. *Acta* 1833, 1078–1084 (2013). 10.1016/j.bbamcr.2013.01.005 23328082

[58] Botchkarev V. A., Gdula M. R., Mardaryev A. N., Sharov A. A., Fessing M. Y., Epigenetic regulation of gene expression in keratinocytes. *J*. *Invest*. *Dermatol*. 132, 2505–2521 (2012). 10.1038/jid.2012.182 22763788 PMC3650472

[59] Kraushaar D. C., Zhao K., The epigenomics of embryonic stem cell differentiation. *Int* . *J*. *Biol*. *Sci*. 9, 1134–1144 (2013). 10.7150/ijbs.7998 PMC3858586 24339734

[60] Tifft K. E., Bradbury K. A., Wilson K. L., Tyrosine phosphorylation of nuclear-membrane protein emerin by Src, Abl and other kinases. *J*. *Cell Sci*. 122, 3780–3790 (2009). 10.1242/jcs.048397 19789182 PMC2758807

[61] Hu Q., Guo C., Li Y., Aronow B. J., Zhang J., LMO7 mediates cell-specific activation of the Rho-myocardin-related transcription factor-serum response factor pathway and plays an important role in breast cancer cell migration. *Mol*. *Cell*. *Biol*. 31, 3223–3240. (2011). 10.1128/MCB.01365-10 21670154 PMC3147800

[62] Wozniak M. A., Baker B. M., Chen C. S., Wilson K. L., The emerin-binding transcription factor Lmo7 is regulated by association with p130Cas at focal adhesions. *PeerJ*. 1, e134 (2013). 10.7717/peerj.134 24010014 PMC3757464

[63] Wu J. I., Diverse functions of ATP-dependent chromatin remodeling complexes in development and cancer. *Acta Biochim*. *Biophys*. *Sin*. 44, 54–69 (2012). 10.1093/abbs/gmr099 22194014

[64] Wilson B. G., Roberts C. W., SWI/SNF nucleosome remodellers and cancer. *Nat*. *Rev*. *Cancer* 11, 481–492 (2011). 10.1038/nrc3068 21654818

[65] Mendoza-Parra M. A., Walia M., Sankar M., Gronemeyer H., Dissecting the retinoid-induced differentiation of F9 embryonal stem cells by integrative genomics. *Mol*. *Sys*. *Biol*. 7, 538 (2011). 10.1038/msb.2011.73 PMC3261707 21988834

[66] Paragh G., Schling P., Ugocsai P., Kel A. E., Liebisch G., Heimerl S., Moehle C., Schiemann Y., Wegmann M., Farwick M., Wikonkál N. M., Mandl J., Langmann T., Schmitz G., Novel sphingolipid derivatives promote keratinocyte differentiation. *Exp*. *Dermatol*. 17, 1004–1016 (2008). 10.1111/j.1600-0625.2008.00736.x 18631249

[67] Hamanaka R. B., Chandel N. S., Mitochondrial metabolism as a regulator of keratinocyte differentiation. *Cell*. *Logist*. 3, e25456 (2013). 24475371 10.4161/cl.25456 PMC3891634

[68] Diss G., Dube A. K., Boutin J., Gagnon-Arsenault I., Landry C. R., A systematic approach for the genetic dissection of protein complexes in living cells. *Cell Rep*. 3, 2155–2167 (2013). 10.1016/j.celrep.2013.05.004 23746448

[69] Csete M., Doyle J., Bow ties, metabolism and disease. *Trends Biotechnol*. 22, 446–450 (2004). 15331224 10.1016/j.tibtech.2004.07.007

[70] Oda K., Matsuoka Y., Funahashi A., Kitano H., A comprehensive pathway map of epidermal growth factor receptor signaling. *Mol*. *Sys*. *Biol* 1, 20050010 (2005). 10.1038/msb4100014 PMC1681468 16729045

[71] Deeds E. J., Krivine J., Feret J., Danos V., Fontana W., Combinatorial complexity and compositional drift in protein interaction networks. *PloS One* 7, e32032 (2012). 10.1371/journal.pone.0032032 22412851 PMC3297590

[72] Suderman R., Deeds E. J., Machines vs. ensembles: effective MAPK signaling through heterogeneous sets of protein complexes. *PLoS Comput*. *Biol*. 9, e1003278 (2013). 10.1371/journal.pcbi.1003278 24130475 PMC3794900

[73] Zhang Q.C., Petrey D., Deng L., Qiang L., Shi Y., Thu C.A., Bisikirska B., Lefebvre C., Accili D., Hunter T., Maniatis T., Califano A., Honig B., Structure-based prediction of protein-protein interactions on a genome-wide scale. *Nature* 490, 556–560 (2012). 10.1038/nature11503 23023127 PMC3482288

[74] Shoemaker B. A., Zhang D., Thangudu R.R., Tyagi M., Fong J.H., Marchler-Bauer A., Bryant S.H., Madej T., Panchenko A.R., Inferred Biomolecular Interaction Server—a web server to analyze and predict protein interacting partners and binding sites. *Nucleic Acids Res*. 38, D518–524 (2010). 10.1093/nar/gkp842 19843613 PMC2808861

[75] Schaefer M. H., Yang J. S., Serrano L., Kiel C., Protein conservation and variation suggest mechanisms of cell type-specific modulation of signaling pathways. *PLoS Comput*. *Biol*. 10, e1003659 (2014). 10.1371/journal.pcbi.1003659 24922536 PMC4055412

[76] Maslov S., Ispolatov I., Propagation of large concentration changes in reversible protein-binding networks. *Proc*. *Natl*. *Acad*. *Sci*. *U*. *S*. *A*. 104, 13655–13660 (2007). 17699619 10.1073/pnas.0702905104 PMC1959437

[77] Romano D., Nguyen L.K., Matallanas D., Halasz M., Doherty C., Kholodenko B.N., Kolch W., Protein interaction switches coordinate Raf-1 and MST2/Hippo signalling. *Nat*. *Cell Biol*. 16, 673–684 (2014). 10.1038/ncb2986 24929361

[78] Pan L., Wang S., Lu T., Weng C., Song X., Park J.K., Sun J., Yang Z.H., Yu J., Tang H., McKearin D.M., Chamovitz D.A., Ni J., Xie T., Protein competition switches the function of COP9 from self-renewal to differentiation. *Nature* 514, 233–236 (2014). 10.1038/nature13562 25119050

[79] Nishi H., Demir E., Panchenko A. R., Crosstalk between Signaling Pathways Provided by Single and Multiple Protein Phosphorylation Sites. *J*. *Mol*. *Biol*. 427, 511–520 (2015). 10.1016/j.jmb.2014.11.001 25451034 PMC4297578

[80] Ritchie M. E., Silver J., Oshlack A., Holmes M., Diyagama D., Holloway A., Smyth G. K., A comparison of background correction methods for two-colour microarrays. *Bioinformatics* 23, 2700–2707 (2007). 17720982 10.1093/bioinformatics/btm412

[81] Bolstad B. M., Irizarry R. A., Astrand M., Speed T. P., A comparison of normalization methods for high density oligonucleotide array data based on variance and bias. *Bioinformatics* 19, 185–193 (2003). 12538238 10.1093/bioinformatics/19.2.185

[82] Stein A., Russell R. B., Aloy P., 3did: interacting protein domains of known three-dimensional structure. *Nucleic Acids Res*. 33, D413–417 (2005). 15608228 10.1093/nar/gki037 PMC539991

[83] Aloy P., Russell R. B., InterPreTS: protein interaction prediction through tertiary structure. *Bioinformatics* 19, 161–162 (2003). 12499311 10.1093/bioinformatics/19.1.161

[84] Edgar R.C., MUSCLE: multiple sequence alignment with high accuracy and high throughput. *Nucleic Acids Res*. 32, 1792–1797 (2004). 15034147 10.1093/nar/gkh340 PMC390337

[85] Shirakihara T., Horiguchi K., Miyazawa K., Ehata S., Shibata T., Morita I., Miyazono K., Saitoh M., TGF-beta regulates isoform switching of FGF receptors and epithelial-mesenchymal transition. *EMBO J*. 30, 783–795 (2011). 21224849 10.1038/emboj.2010.351 PMC3041949

[86] Schymkowitz J., Borg J., Stricher F., Nys R., Rousseau F., Serrano L., The FoldX web server: an online force field. *Nucleic Acids Res*. 33, W382–388 (2005). 15980494 10.1093/nar/gki387 PMC1160148

[87] Needleman S. B., Wunsch C. D., A general method applicable to the search for similarities in the amino acid sequence of two proteins. *J*. *Mol*. *Biol*. 48, 443–453 (1970). 5420325 10.1016/0022-2836(70)90057-4

