# Supplementary figures and images for: Dissecting the Calcium-Induced Differentiation of Human Primary Keratinocytes Stem Cells by Integrative and Structural Network Analyses
Source: PLoS Comput Biol. 2015 May 6;11(5):e1004256. doi: 10.1371/journal.pcbi.1004256 (PMC4422705; doi:10.1371/journal.pcbi.1004256)

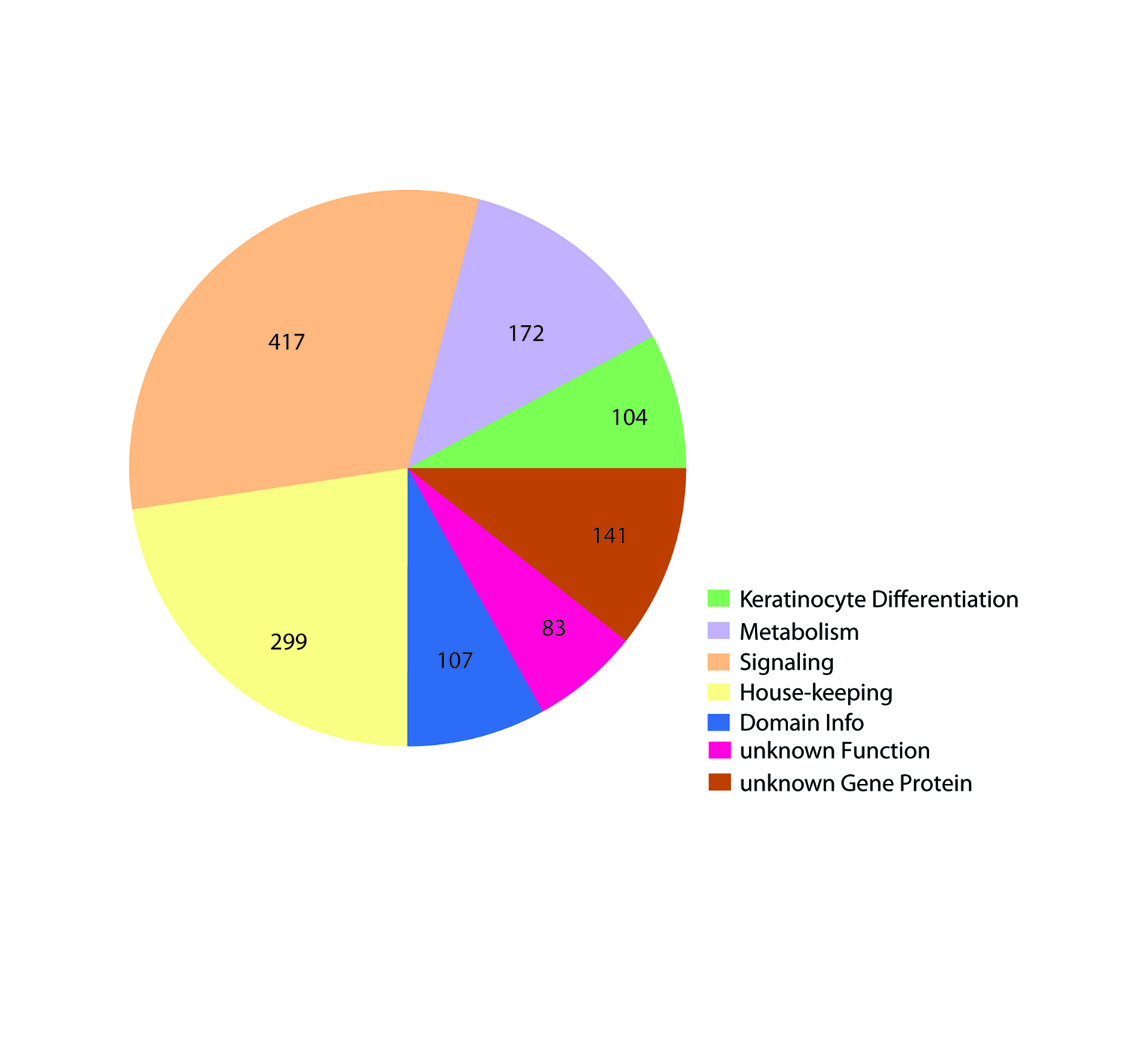

Supplement: S1 Fig — Classification was based on DAVID, UniProt, and manual literature searches into proteins needed for the formation of the cornified envelope (‘Keratinocyte Differentiation’), Metabolism, general signaling, and housekeeping. For some proteins only a Pfam domain prediction can be assigned (‘Domain Info’), or have no domain and an unknown function (‘unknown Function). The remaining proteins are not annotated (‘unknown Gene Protein’). (TIF) [file pcbi.1004256.s001.tif]

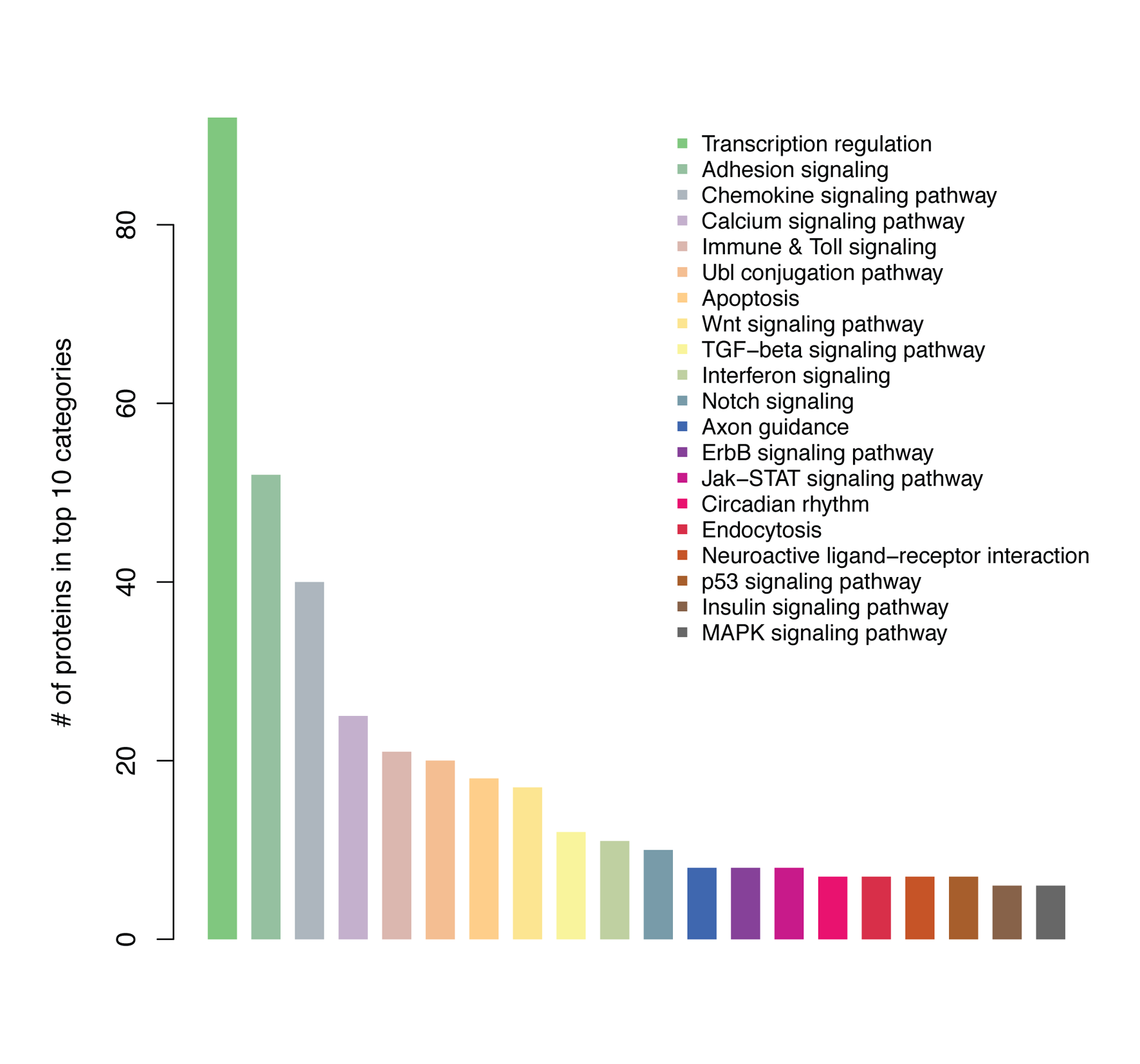

Supplement: S2 Fig — Further functional sub-classification of the proteins related to signaling based on DAVID, UniProt, and manual literature searches. (TIF) [file pcbi.1004256.s002.tif]

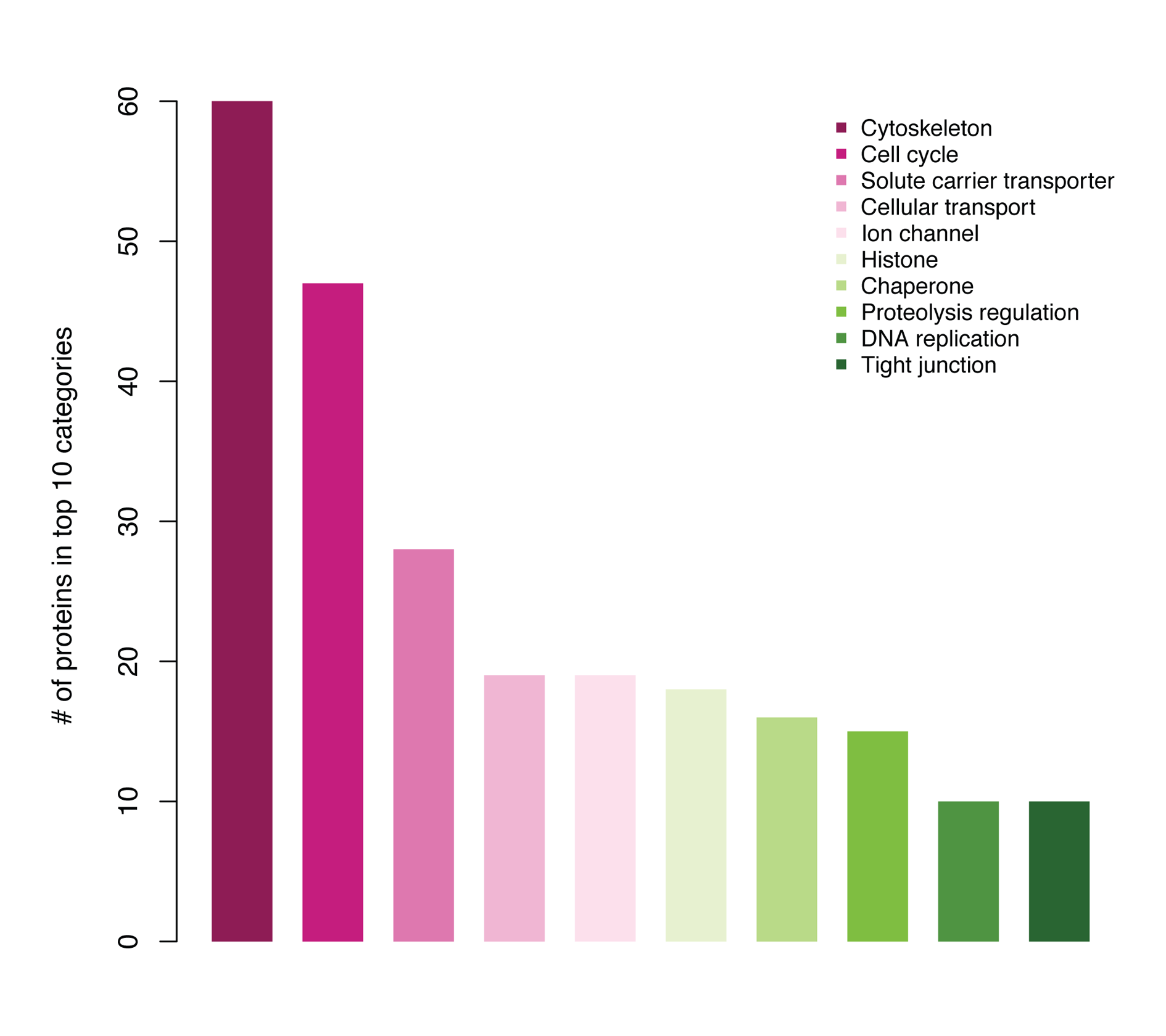

Supplement: S3 Fig — Further functional sub-classification of the housekeeping proteins based on DAVID, UniProt, and manual literature searches. (TIF) [file pcbi.1004256.s003.tif]

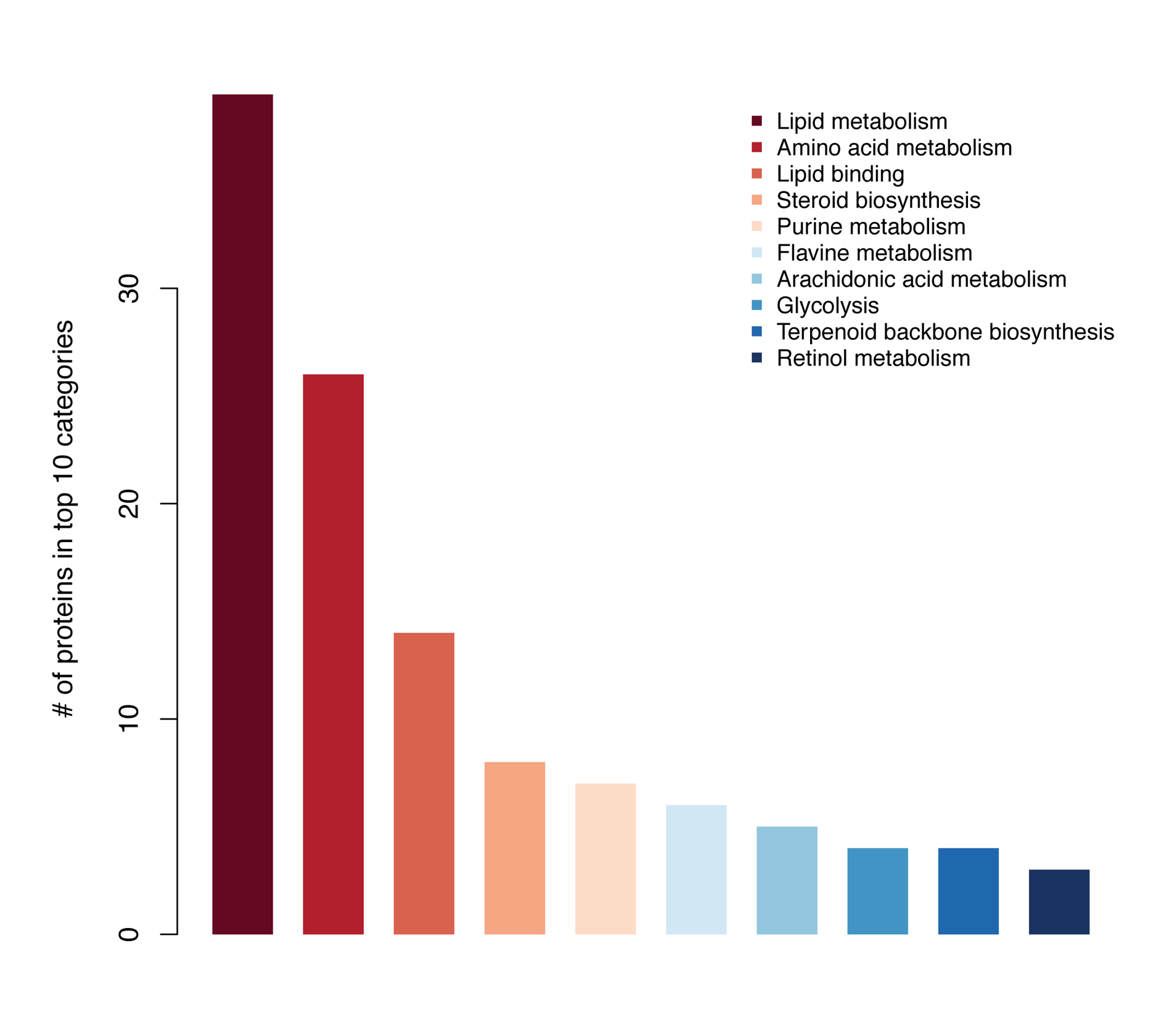

Supplement: S4 Fig — Further functional sub-classification of the proteins related to metabolism based on DAVID, UniProt, and manual literature searches. (TIF) [file pcbi.1004256.s004.tif]

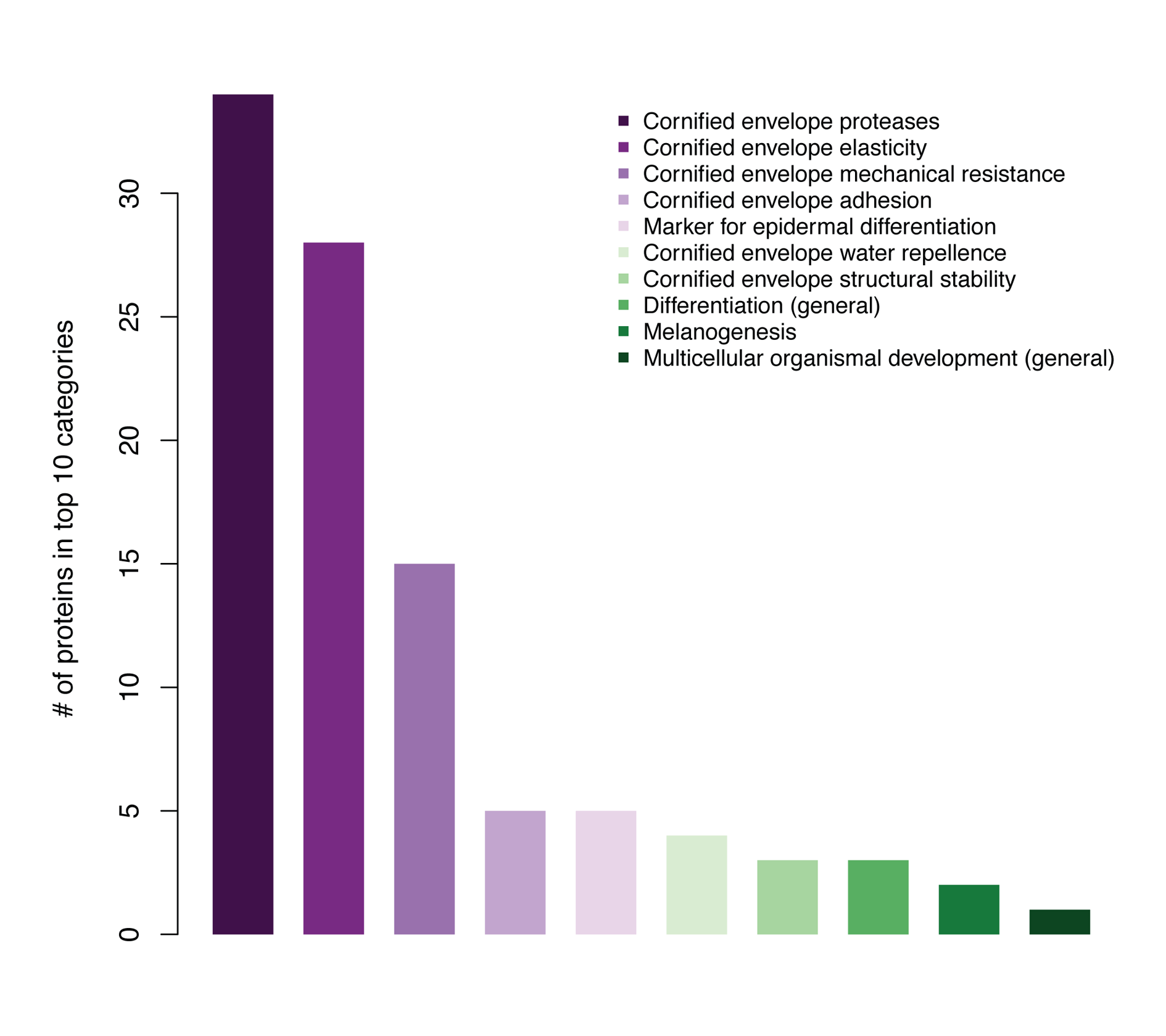

Supplement: S5 Fig — Further functional sub-classification of the proteins related to keratinocyte differentiation (‘Keratinocyte Differentiation’) based on DAVID, UniProt, and manual literature searches. (TIF) [file pcbi.1004256.s005.tif]

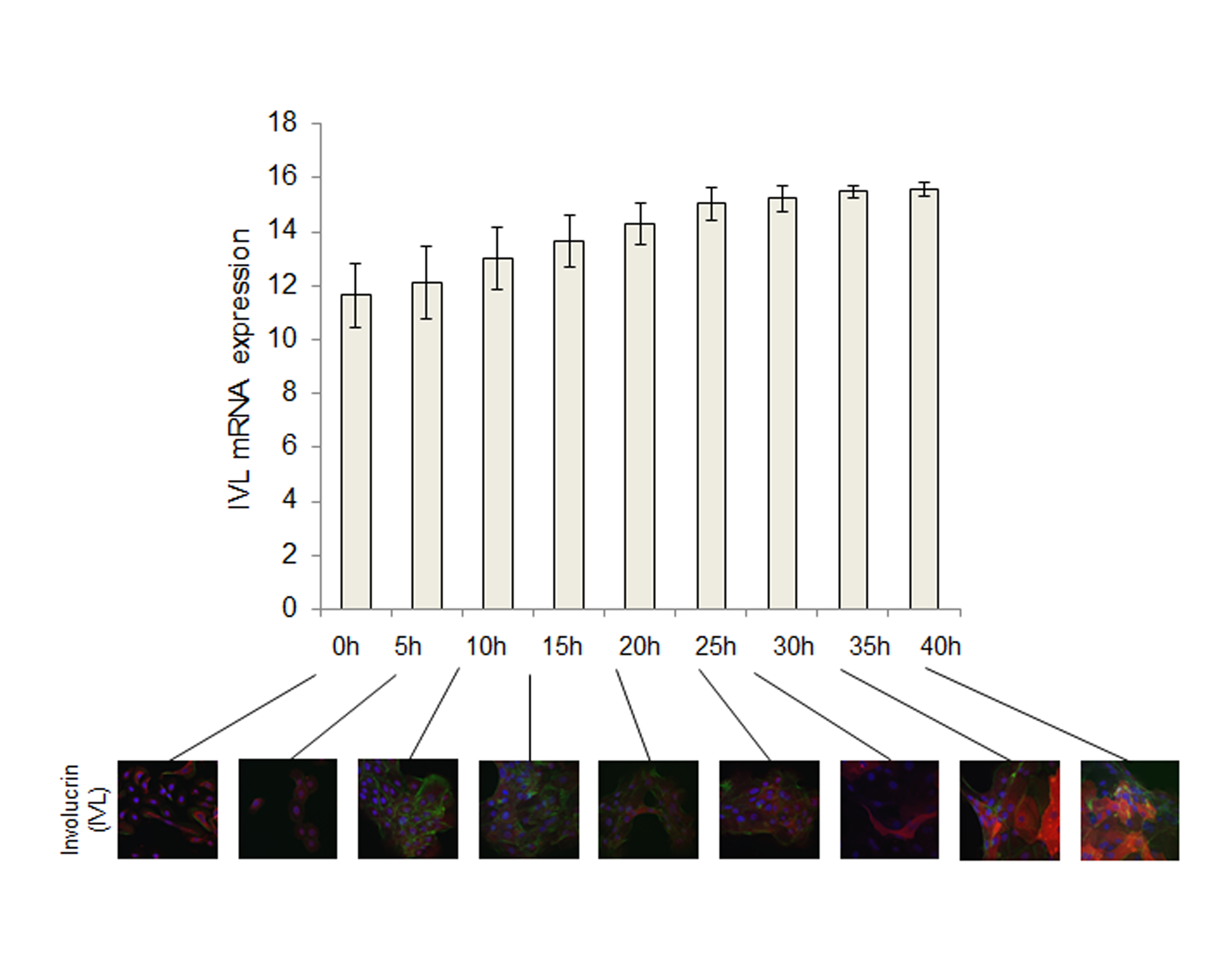

Supplement: S6 Fig — Comparing gene expression changes (bar diagrams) with protein expression of involucrin (IVL) by immunostaining (inserted pictures) at different time points after calcium treatment. (TIF) [file pcbi.1004256.s006.tif]

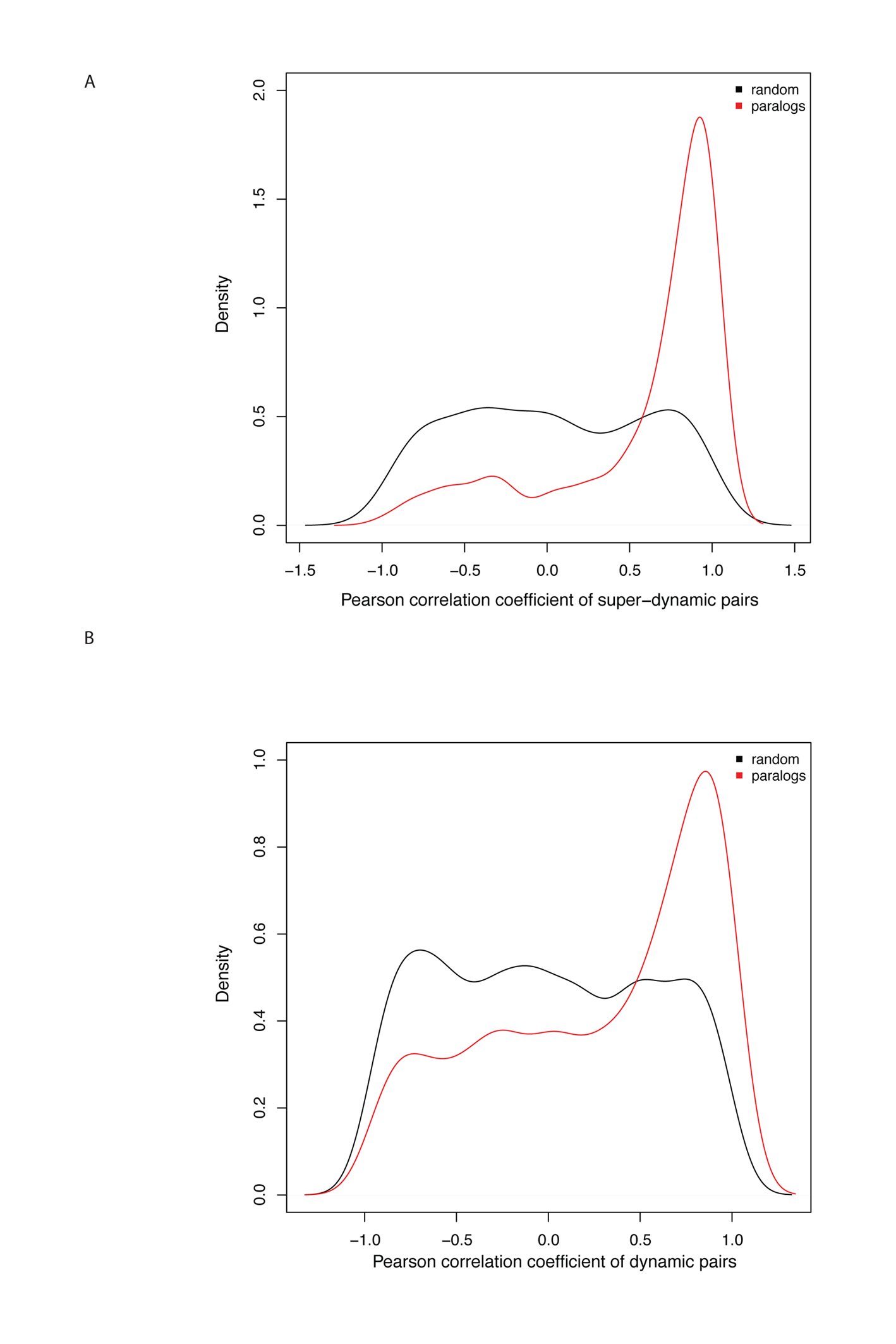

Supplement: S7 Fig — Density plots of Pearson correlation coefficients (PCC) of gene expression comparison of (A) paralog super-dynamic gene pairs (n = 340, mean = 0.6, median = 0.8) to random super-dynamic gene pairs (n = 340, mean = 0, median = 0) indicates a real shift in distributions (P = 4.5e−37) and also (B) paralog dynamic gene pairs (n = 2,260, mean = 0.3, median = 0.4) to random dynamic gene pairs (n = 2,260, mean = 0, median = 0) indicates a real shift in distributions (P = 5.4e−61). Statistical significance tested using Wilcoxon rank sum test. (TIF) [file pcbi.1004256.s007.tif]

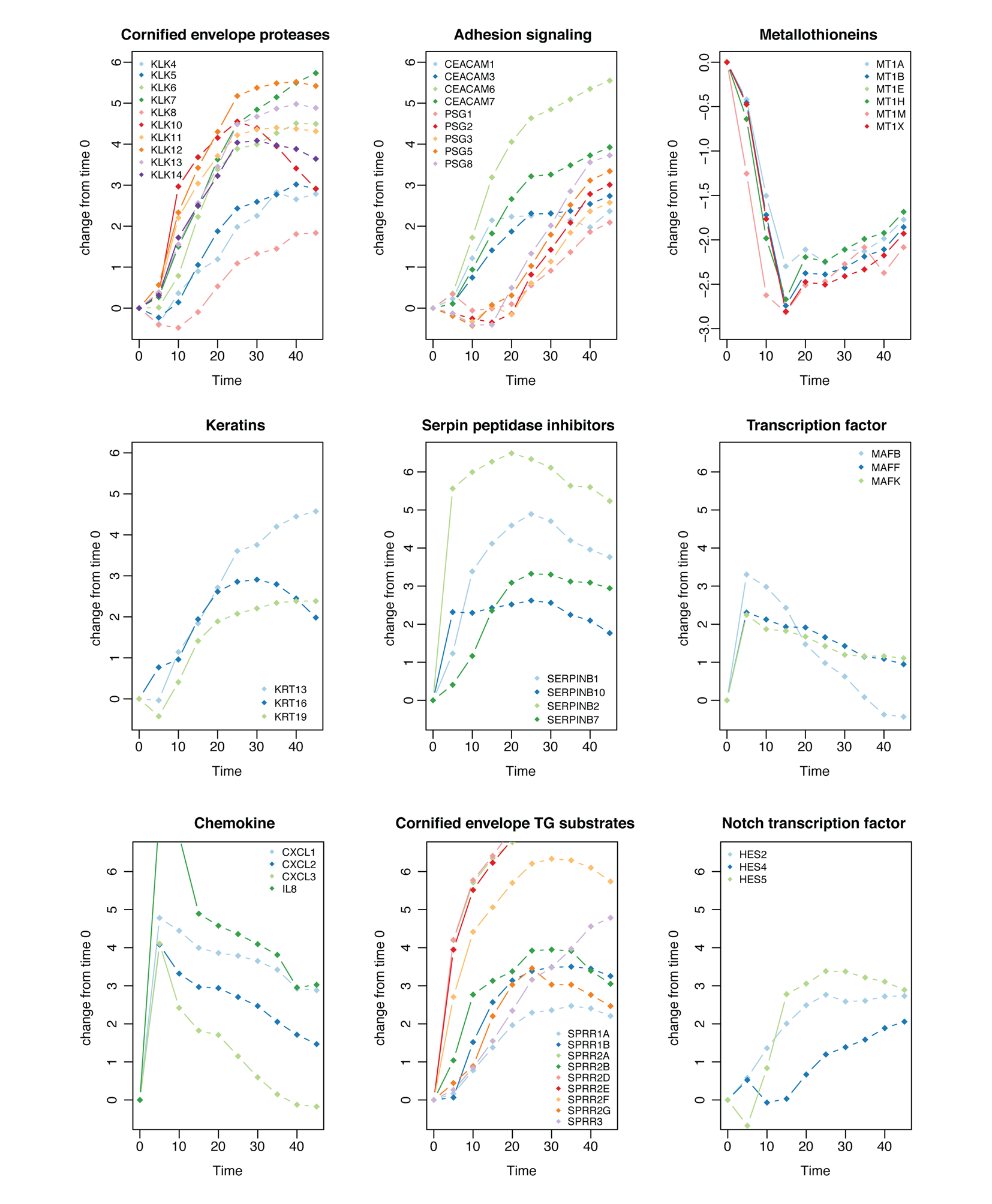

Supplement: S8 Fig — The expression plotted compared to time 0 against time after calcium induction (log transformed data). (TIF) [file pcbi.1004256.s008.tif]

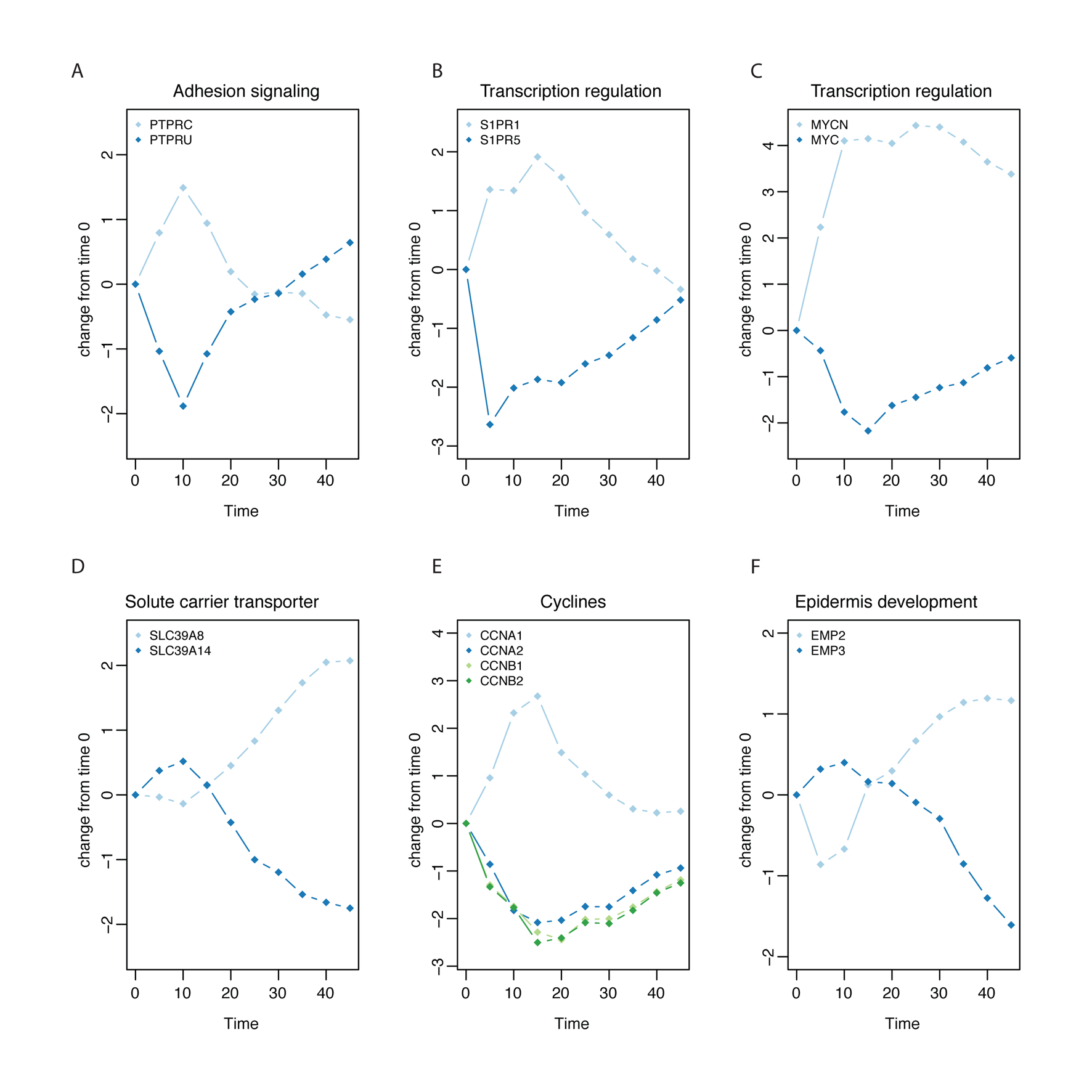

Supplement: S9 Fig — The expression plotted compared to time 0 against time after calcium induction (log transformed data). (TIF) [file pcbi.1004256.s009.tif]

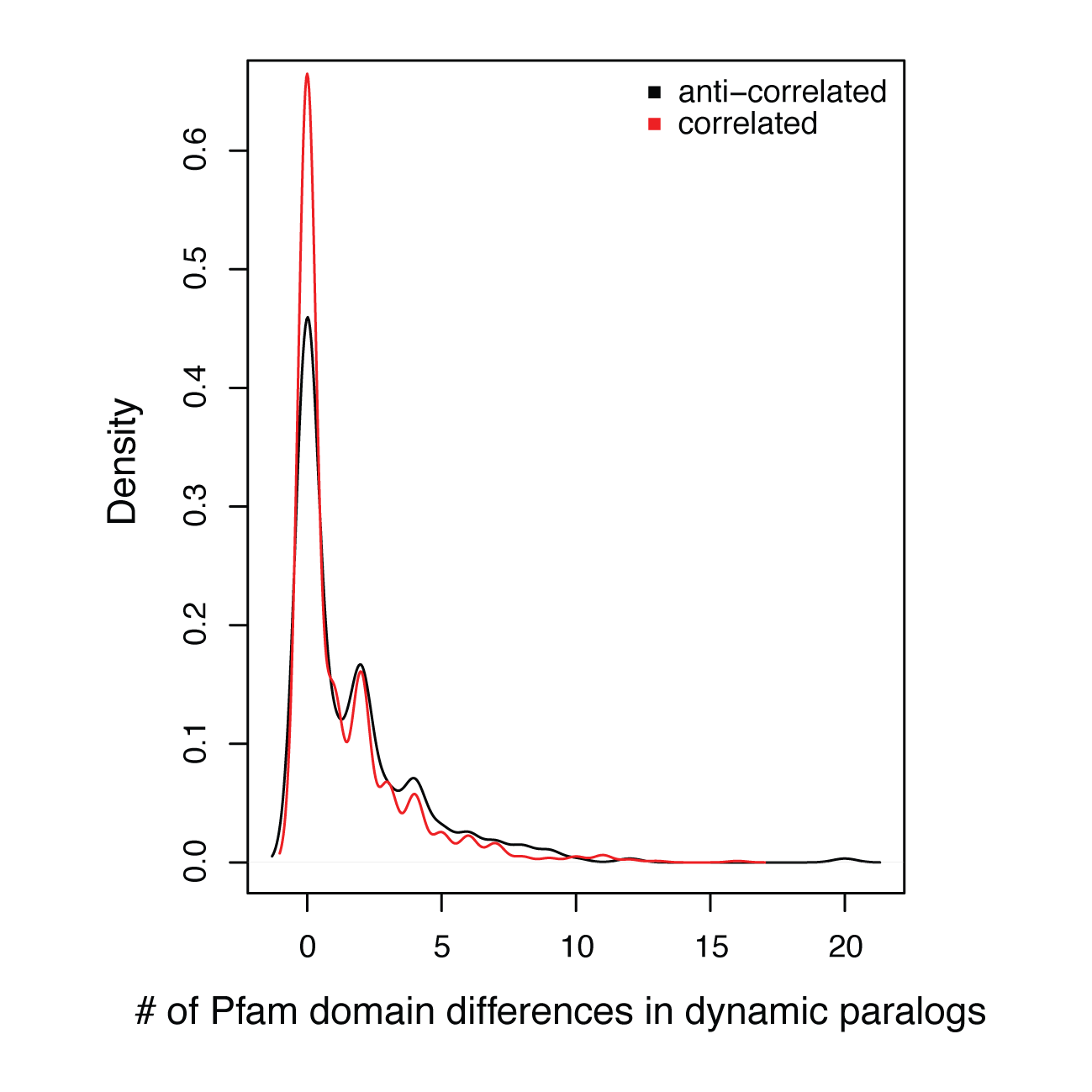

Supplement: S10 Fig — Dynamic anti-correlated paralogs (n = 281, r< = −0.6) have a mean of 1.7 differences in the total number of domains compared to dynamic correlated paralogs (n = 953, r> = 0.6) which have a mean of 1.3 total differences in how many Pfam domains they have (P = 0.008). (TIF) [file pcbi.1004256.s010.tif]

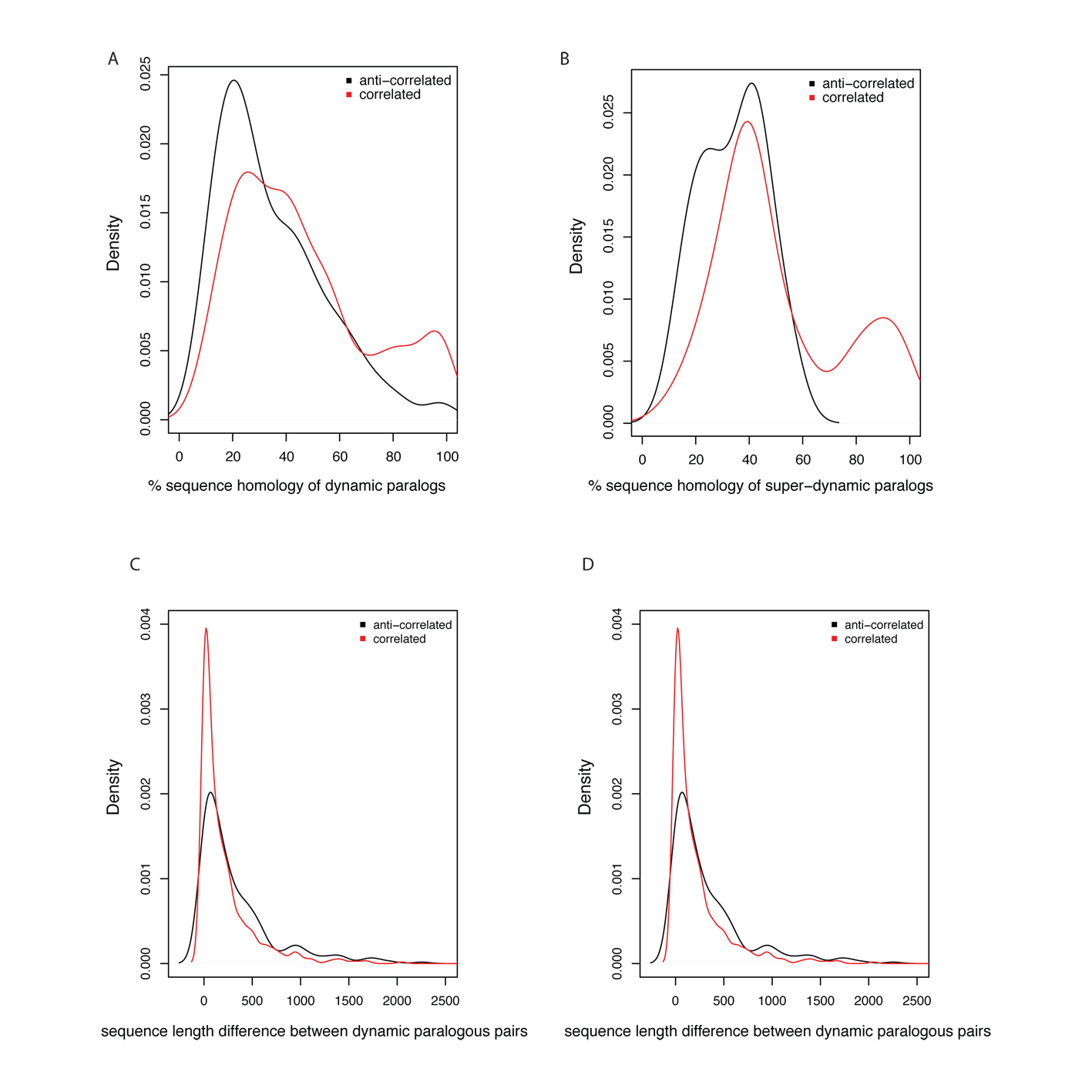

Supplement: S11 Fig — (A) Dynamic anti-correlated paralogs (n = 281, r< = -0.6) have on average 34% sequence homology compared to dynamic correlated paralogs (n = 949, r> = 0.6) which are on average 46% similar at sequence level (P = 3.8e−11). (B) Super-dynamic anti-correlated paralogs (n = 19, r< = −0.6) have on average 34% sequence homology compared to 51% for super-dynamic correlated paralogs (n = 234, r> = 0.6) (P = 0.005). (C) Dynamic anti−correlated paralog pairs (n = 281, r< = −0.6) have an average 330 residue-difference in their sequence length compared to correlated paralog pairs (n = 952,r> = 0.6) which have an average sequence length difference of 227 residues (P = 8.3e−08). (D) Super-dynamic anti−correlated paralog pairs (n = 19, r< = −0.6) have a mean sequence length difference of 247 residues compared to correlated paralogs (n = 235, r> = 0.6) which have a mean difference of 128 (P = 0.01). Statistical significance tested using Wilcoxon rank sum test. (TIF) [file pcbi.1004256.s011.tif]

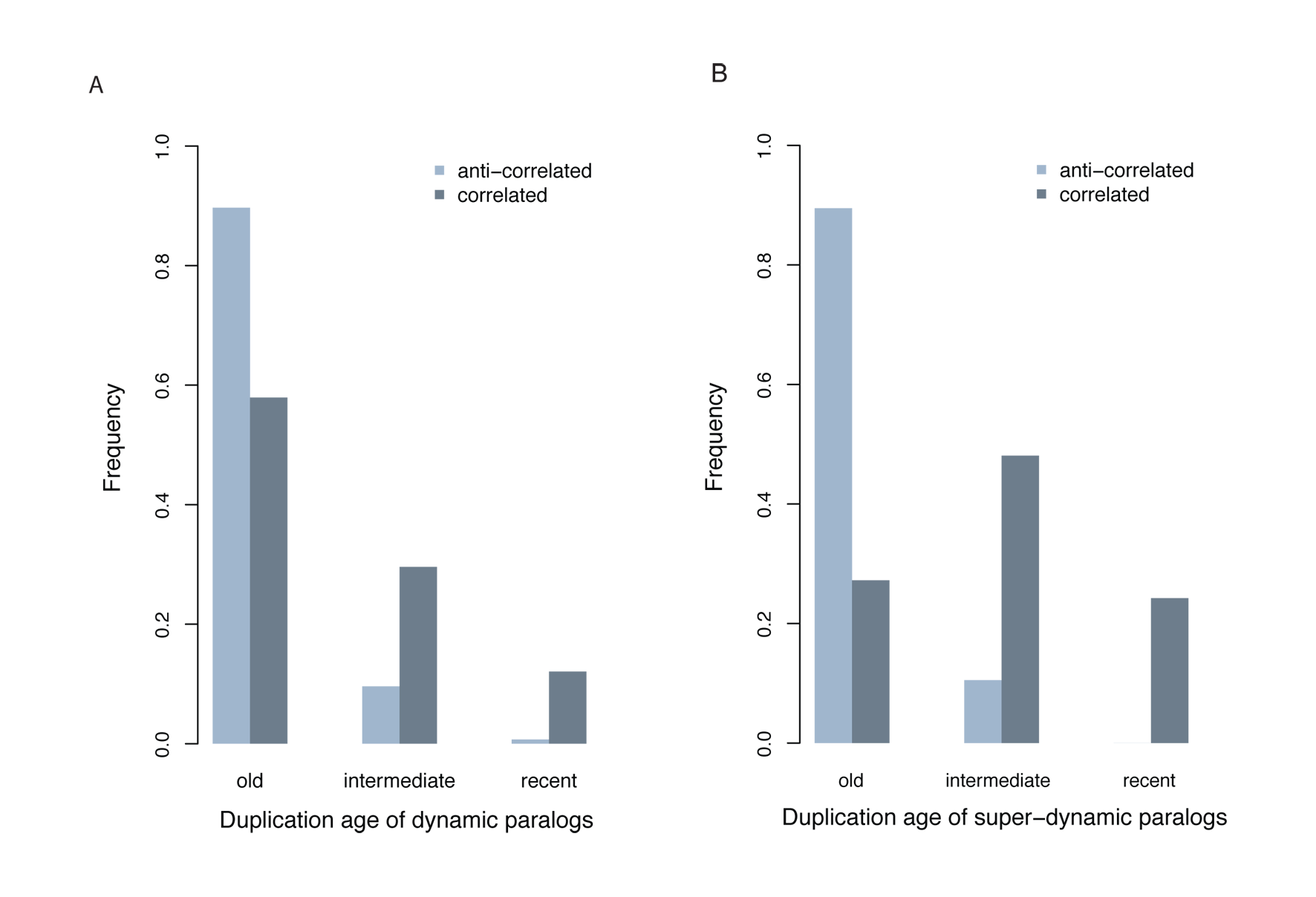

Supplement: S12 Fig — (A) comparison of dynamic anti-correlated paralog pairs (n = 281, r< = -0.6) to dynamic correlated paralog pairs (n = 953, r> = 0.6) indicates that 90% of the former group are the result of old duplication events compared to 58% of the latter (P = 1.5e−25), 10% are the result of intermediate duplication events compared to 30% (P = 4. 5e−13) and finally 1% are from recent duplication events compared to 12% (1.4e−11). (B) comparison of super-dynamic anti-correlated paralog pairs (n = 19, r< = -0.6) to super-dynamic correlated paralog pairs (n = 235, r> = 0.6) indicates that 90% of the former group are the result of old duplication events compared to 27% of the latter (P = 1.05e−07), 11% are the result of intermediate duplication events compared to 48% (P = 0.001), and lastly 0% are from recent duplication events compared to 24% (P = 0.009). Statistical significance tested using Fisher's exact test. (TIF) [file pcbi.1004256.s012.tif]

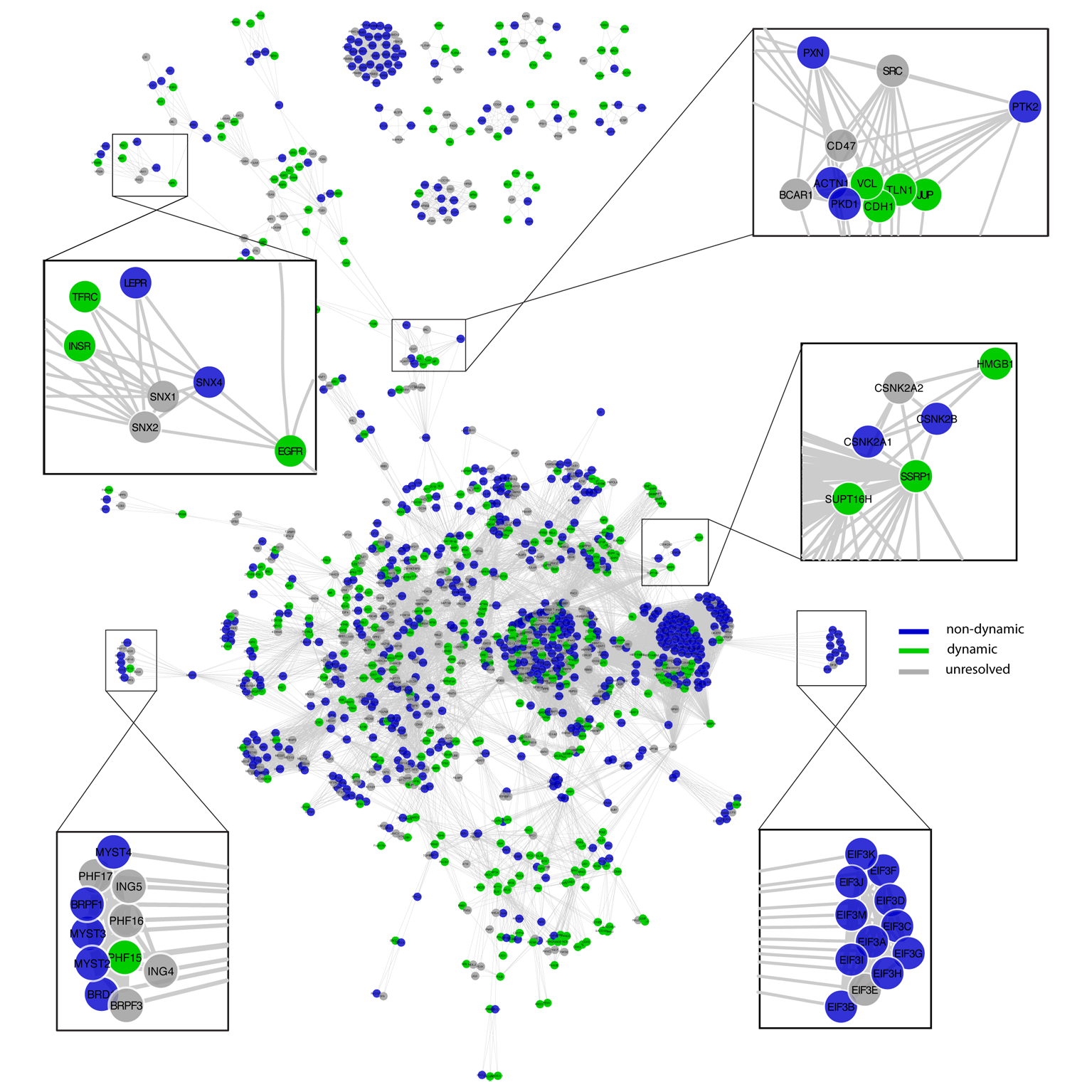

Supplement: S13 Fig — Protein expression changes mapped on the human complexes from CORUM. The node color represents the expression category (see legend). Same pattern observed by others before emerges in the global map whereby complexes are usually a mix of both non-dynamically and dynamically expressed genes. About 20% of protein complexes showed statistically significant (p. < 0.05) co-regulation in the expression profiles of their subunits. Similarly a large number of protein families and pathways showed concerted temporal dynamics among their components (S3 Table). Complexes are highly inter-connected. (TIF) [file pcbi.1004256.s013.tif]

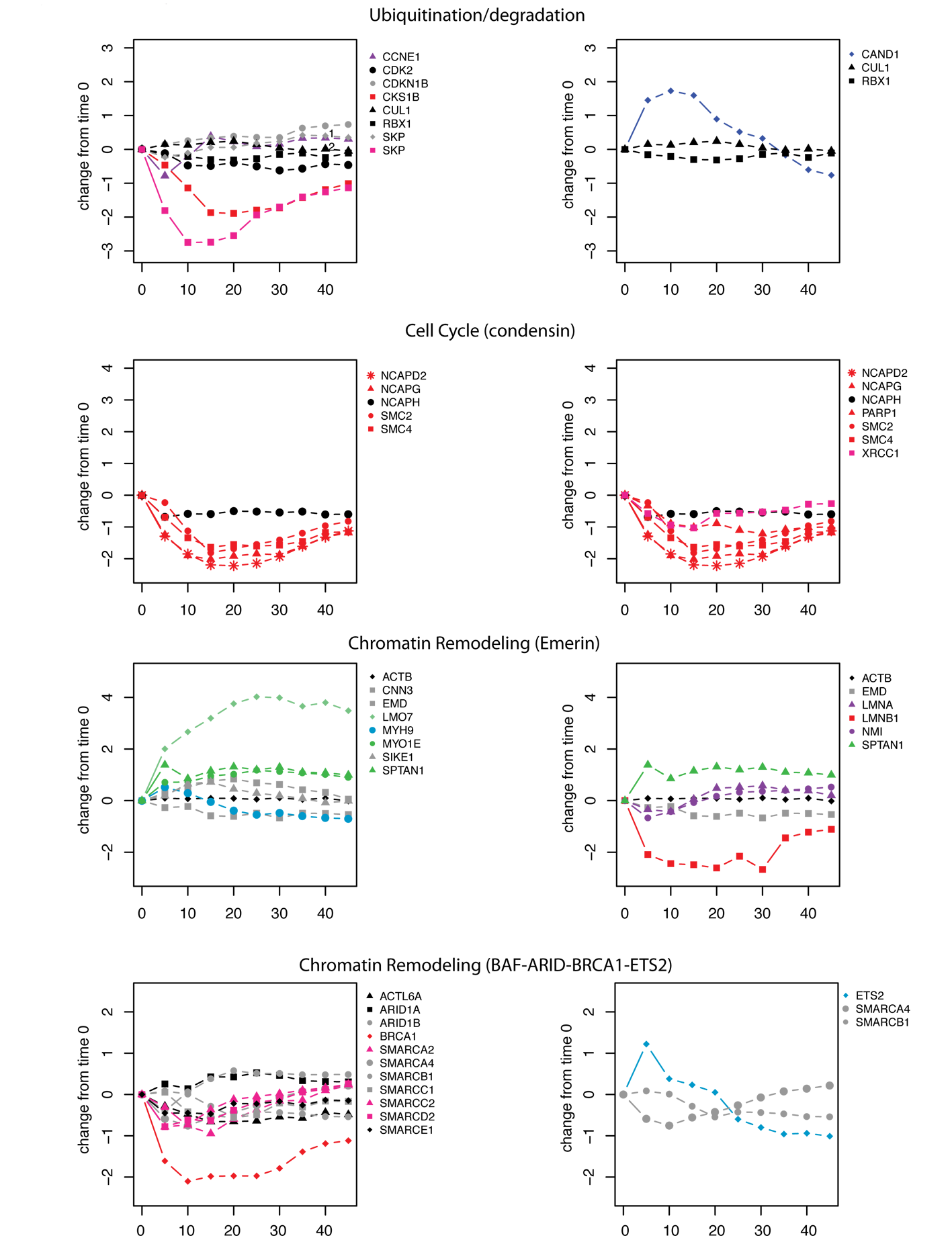

Supplement: S14 Fig — Selected CORUM complexes of similar core proteins were plotted. Super-dynamic and dynamic genes were colored according to clusters. Black indicates constitutive genes, grey indicated unresolved genes. (TIF) [file pcbi.1004256.s014.tif]

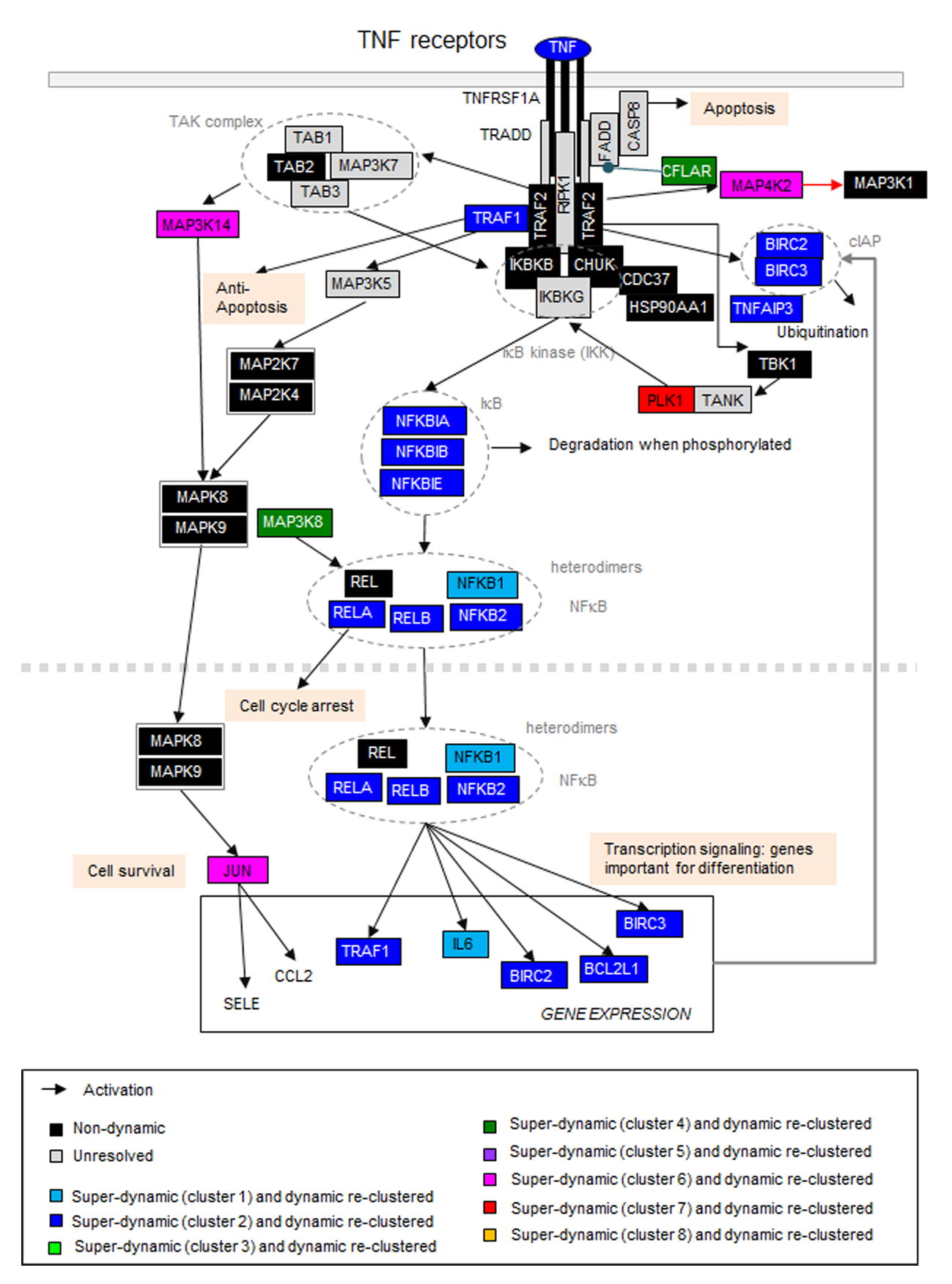

Supplement: S15 Fig — The manually-curated network is based on [44] and [45]. Genes are colored based on expression classification (see legend). TNFRSF1A is the TNF receptor with broad tissue expression. Other TNF receptor and TRAF family members are displayed in boxes on the left side. See legend for details. (TIF) [file pcbi.1004256.s015.tif]

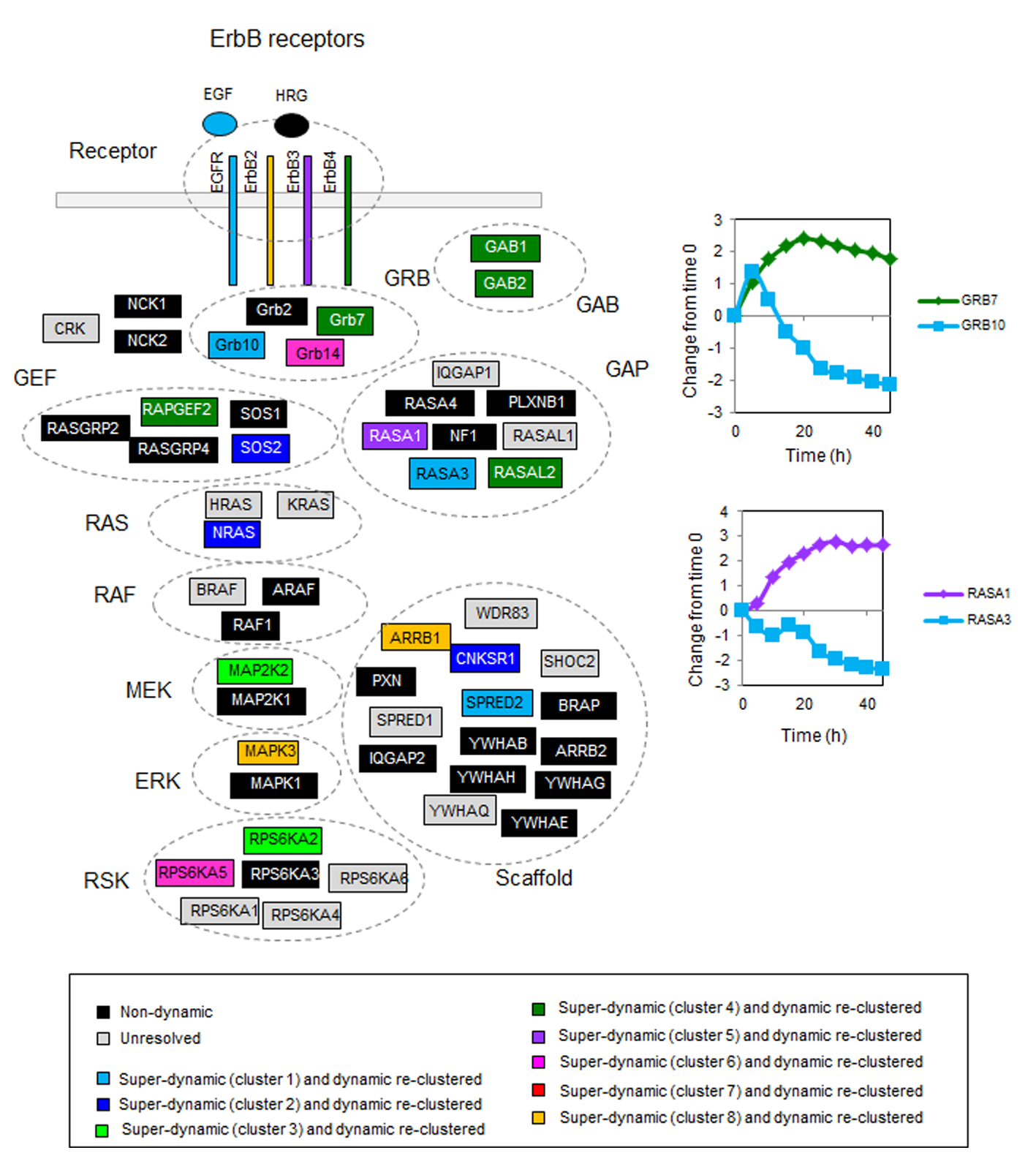

Supplement: S16 Fig — Genes are colored based on expression classification (see legend). (TIF) [file pcbi.1004256.s016.tif]

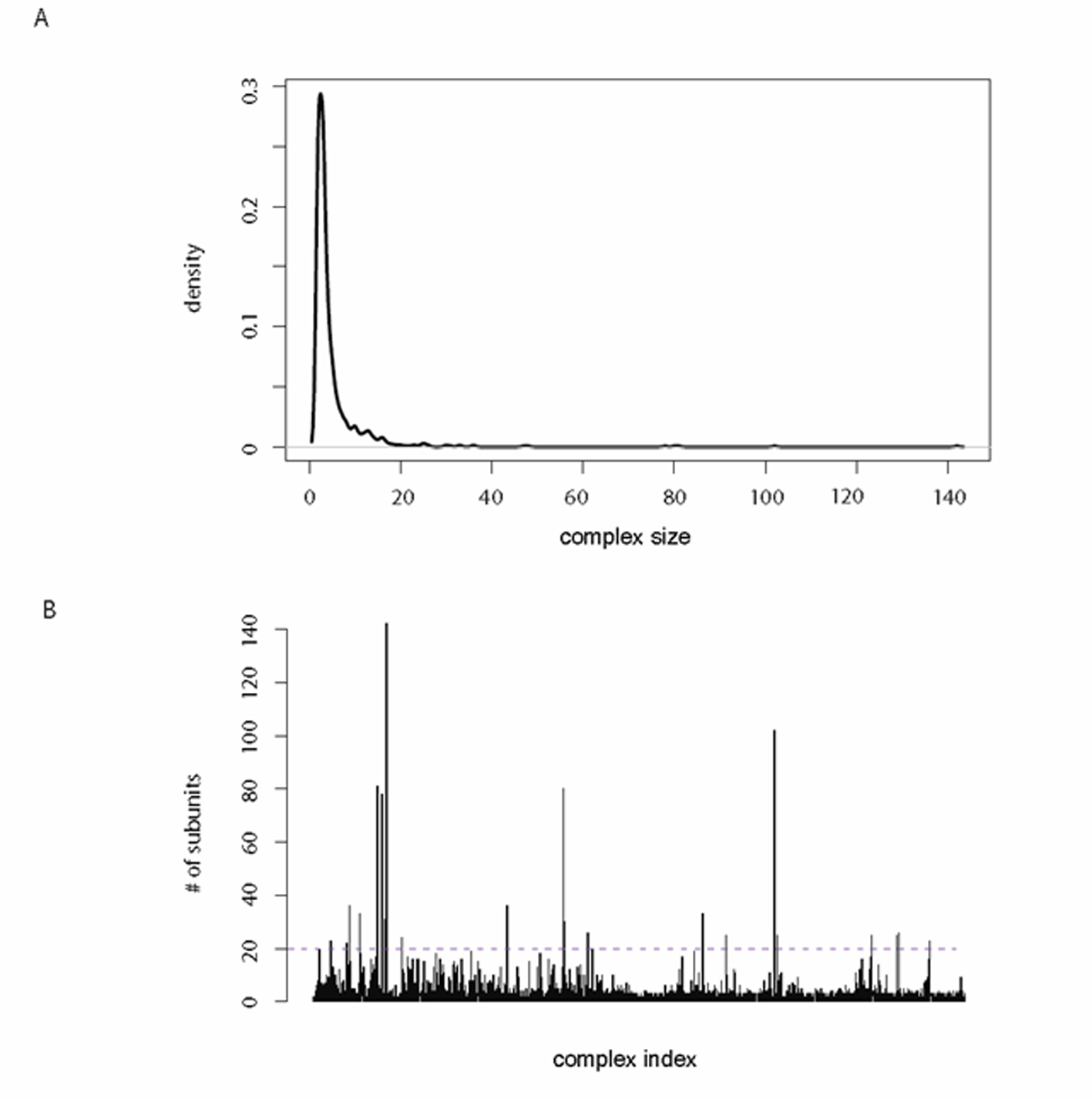

Supplement: S17 Fig — (A) Distribution of protein complex sizes (number of subunits) indicates that the majority of complexes have less than 20 subunits. (B) We impose a threshold of 20 on complex size for the downstream SAPIN-mediated structural analyses (S4 Table). (TIF) [file pcbi.1004256.s017.tif]

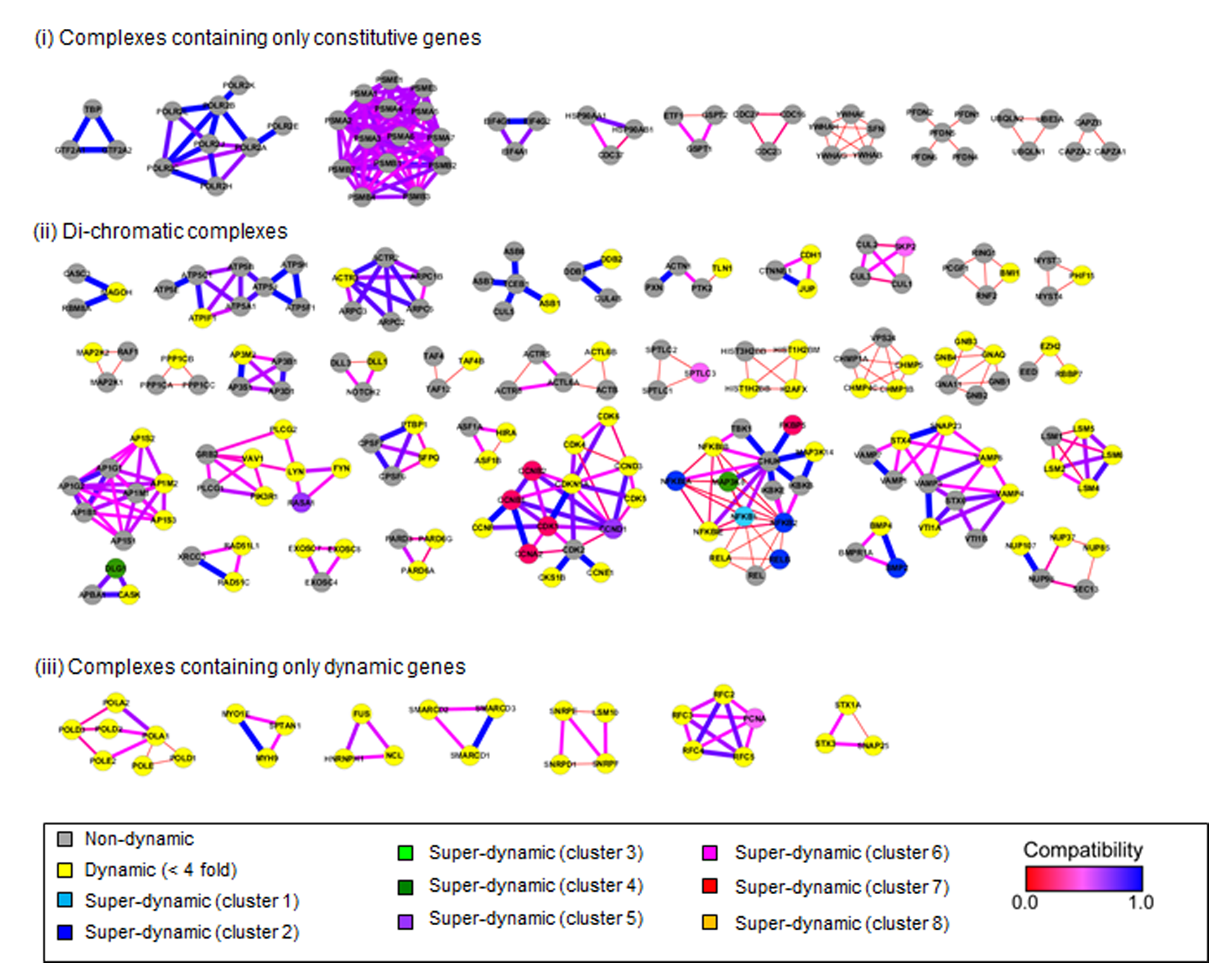

Supplement: S18 Fig — Network representation of compatibility scores calculated using SAPIN. We show only non-dynamic and dynamic cases and not unresolved cases. The edge color and width corresponds to the average surface compatibility. The nodes are Nodes colored according to expression or and cluster membership. Please see the legend for details. The network is represented using Cytoscape. (TIF) [file pcbi.1004256.s018.tif]

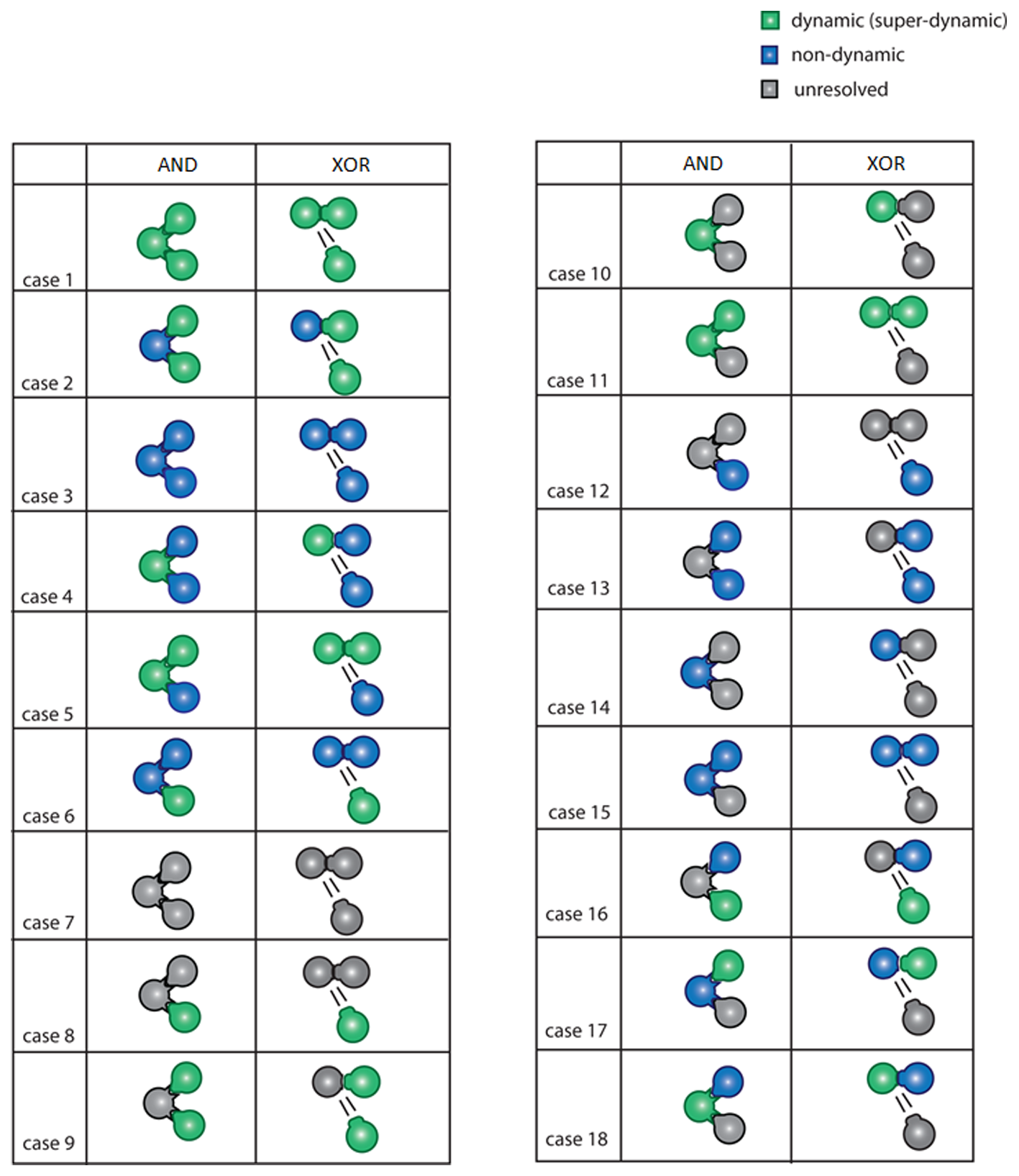

Supplement: S19 Fig — There are a total of 18 possible cases if we take all expression classes—dynamic/super-dynamic, non-dynamic, unresolved—into account. (TIF) [file pcbi.1004256.s019.tif]

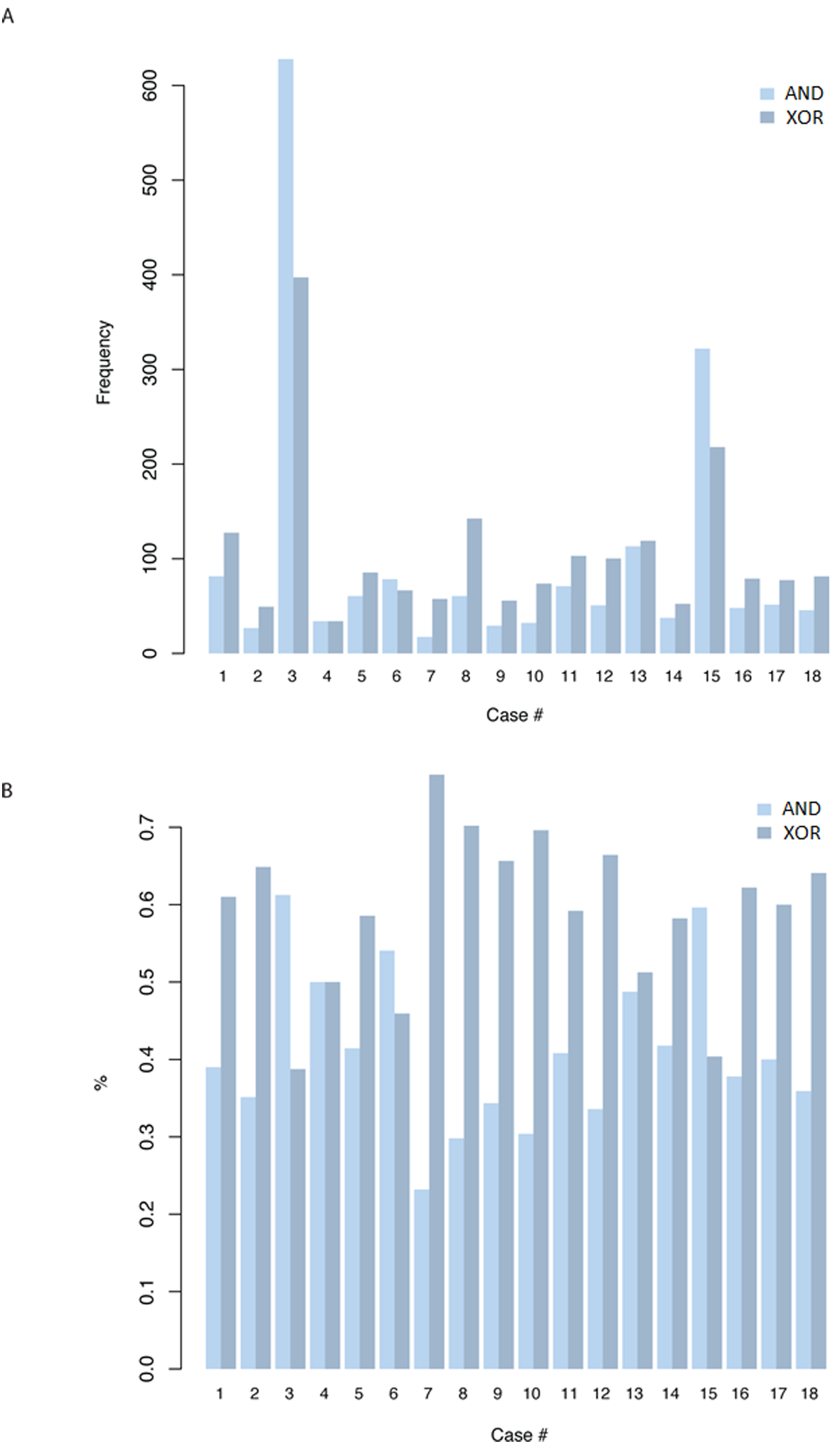

Supplement: S20 Fig — (A) Frequency (count data) comparison of AND and XOR for 18 cases. (B) Percentage (ratios) comparison of AND and XOR for 18 cases. (TIF) [file pcbi.1004256.s020.tif]

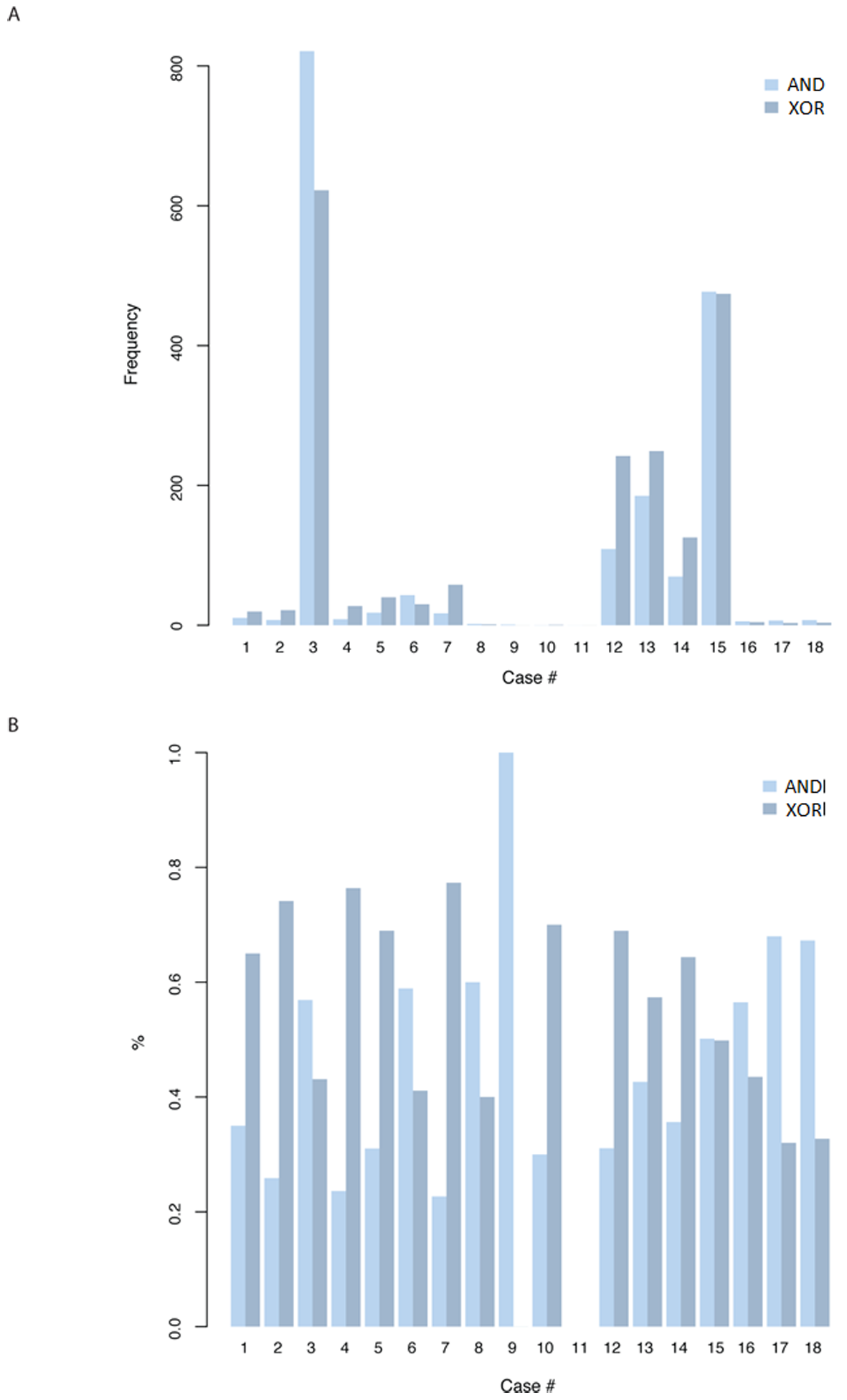

Supplement: S21 Fig — (A) Frequency (count data) comparison of AND and XOR for 18 cases. (B) Percentage (ratios) comparison of AND and XOR for 18 cases. (TIF) [file pcbi.1004256.s021.tif]
